# A Taxonomic and Phylogenetic Contribution on *Inosperma* Section *Inosperma* (*Agaricales*, *Inocybaceae*) in Europe: Calamistratum and Geraniodorum Groups

**DOI:** 10.3390/jof10060374

**Published:** 2024-05-23

**Authors:** Fernando Esteve-Raventós, Ellen Larsson, Fermín Pancorbo, Enrico Bizio, Alberto Altés, Yolanda Turégano, Gabriel Moreno, Ibai Olariaga

**Affiliations:** 1 Departamento de Ciencias de la Vida (Botánica), Facultad de Ciencias, Universidad de Alcalá, 28805 Alcalá de Henares, Madrid, Spain; alberto.altes@uah.es (A.A.); yolandytc@hotmail.com (Y.T.); gabriel.moreno@uah.es (G.M.); 2 Biological and Environmental Sciences and Gothenburg Global Biodiversity Centre, University of Gothenburg, P.O. Box 463, SE 405 30 Göteborg, Sweden; ellen.larsson@bioenv.gu.se; 3 Sociedad Micológica de Madrid, Real Jardín Botánico, C/Claudio Moyano 1, 28014 Madrid, Madrid, Spain; fermin.pancorbo@gmail.com; 4 Società Veneziana di Micologia, Museo Storia Naturale di Venezia Giancarlo Ligabue, Santa Croce 1730, 30135 Venezia, Italy; enrico.bizio@gmail.com; 5 Departamental II, Departamento de Biología, Geología, Física y Química, Universidad Rey Juan Carlos, Despacho 252, 28933 Móstoles, Madrid, Spain; ibai.olariaga@urjc.es

**Keywords:** diversity, *Inocybe* s.l., molecular systematics, new species, phylogeny, taxonomy

## Abstract

The aim of this study is to carry out a taxonomic revision of the groups Calamistratum and Geraniodorum of the genus *Inosperma* sect. *Inosperma* in Europe. For this purpose, a multigenic phylogenetic analysis was carried out using the ITS, LSU, RPB1 and RPB2 markers, covering a total of 111 sequences, including those generated from the existing type-material collections. This analysis led to the recognition of nine clades or terminal groups for the European continent, correlating with nine morphological species. Three of them, *I. calamistratum*, *I. neohirsutum* sp. nov. and *I. turietoense* sp. nov., are distributed in humid and temperate forests, whereas *I. geminum* sp. nov., *I. geraniodorum*, *I. gracilentum* sp. nov., *I. praetermissum* comb. nov., *I. subhirsutum* and *I. veliferum* seem to be restricted to the colder altimontane, boreal and alpine climates. It is concluded that the study of morphological and ecological characteristics allows the recognition of species without the need for an often-subjective interpretation of organoleptic characteristics. *Inocybe hirsuta* is considered a synonym of *Inosperma calamistratum*, *Inosperma praetermissum* as a different species from *I. calamistratum*, and *Inocybe geraniodora* var. *gracilenta* f. *salicis-herbaceae* as a synonym of *I. praetermissum*. Four new species and one new combination are proposed. A key for the recognition of the European species is provided. Illustrations and photographs of macro- and micromorphological characters and SEM spores of all species are presented.

## 1. Introduction

The genus *Inosperma* (Kühner) Matheny & Esteve-Rav. is currently one of the seven genera that form part of the family *Inocybaceae* Jülich [1], a monophyletic and cosmopolitan family, with ectomycorrhizic representatives that live in symbiotic association with a total of 23 vascular plant families [2]. The estimated number of species of *Inocybaceae* worldwide is well over a thousand [3], with Europe and North America being the areas with the best representation and understanding of their diversity.

The species of the genus *Inosperma* are characterised by the absence of pleurocystidia and often a marked reddening of the context and very distinctive odours (earthy, fishy, aromatic, fruity, pelargonium, truffle-like, etc.). More than 55 species are known worldwide, distributed on all continents except South America [2]. Until the recognition of *Inosperma* as a genus, its species were grouped in several sections of the genus *Inocybe* (Fr.) Fr. subgenus *Inosperma* Kühner [4,5]. Among these sections or groups, two are well represented in the Northern Hemisphere and correspond to (1) the Cervicolores group or clade (= *Inocybe* subg. *Inosperma* section *Cervicolores* Kühner & Romagn. ex Singer) and (2) the Maculata group or clade (= *Inocybe* subg. *Inosperma* sect. *Rimosae* (Fr.) Quél. sensu Larsson et al. 2009 pro parte). The genus *Inosperma* also includes two evolutionary lineages that are still poorly known in tropical Asia and Africa [2].

*Inosperma* is widely represented in Europe and North America, comprising about 35–40 species [2], while about 15 more have been described from Asia and Australasia, according to Latha and Manimohan [6] and Matheny and Bougher [7]. The Cervicolores group/clade is represented by species characterised by a usually very pronounced reddening of the context; a fibrillose, woolly, tomentose, scaly or strigose pileus; and very slender (Q > 4.0) and necropigmented basidia. The Maculatae group/clade includes species which differ greatly in appearance from the former, as they usually show little or no reddening at all, the pileus is smooth and radially fibrous to rimose, and the basidia lack necropigment and are not as slender (Q < 4.0). The Maculatae were traditionally considered to be part of the section *Rimosae* of *Inocybe* subg. *Inosperma* [4,5,8] due to the smooth, rimose appearance of the pileus and their hardly reddening flesh. However, following the study of Matheny et al. [2], based on a six-gene phylogeny, it has been shown that the traditional section *Rimosae* should be integrated into the genus *Pseudosperma* Matheny & Esteve-Rav., as its species are phylogenetically rather distant from *Inosperma*. Bizio [9] offers a detailed historical review of the different treatments of these two groups of *Inocybe*.

In this contribution, we present a taxonomic and phylogenetic study of part of the *Inosperma* species present in Europe, specifically two groups that we have named Calamistratum and Geraniodorum. Both have traditionally been part of *Inocybe* sect. *Cervicolores*, which was proposed invalidly by Kühner and Romagnesi [10] and later validated by Singer [11]. The same treatment was subsequently adopted by Kuyper [4] in his European monograph on the smooth-spored *Inocybe*. As mentioned above, based on the phylogenetic study by Matheny et al. [2], the Cervicolores should now correspond to the section *Inosperma* of the genus *Inosperma* with the type species *Inosperma calamistratum* (Fr.) Matheny & Esteve-Rav.

Bon [5] considered two groups or tribes (Bongardii and Calamistrata) for the Cervicolores, which, according to the author, can be distinguished by the intensity of the flesh reddening, the colour of the basidiomata and the spore Q-value. Most species of the Calamistratum group can be easily recognised macroscopically by the blue-greenish shades on the stipe and the strigose–squamose appearance of the pileus surface; on the other hand, the species of the Geraniodorum group lack this striking colouration and are characterised by their small to very small size, a distinctive pelargonium or geranium-like odour, their preference for high mountain and boreal/alpine habitats, and the fibrillose, sometimes subsquamose appearance of the pileus. In this respect, the Geraniodora members may be reminiscent of other *Inosperma* species, referred to here as the Cervicolor group, containing well-known species like *Inosperma cervicolor* (Pers.) Matheny & Esteve-Rav. and *I. bongardii* (Weinm.) Matheny & Esteve-Rav., but these generally have a larger size, thrive in more mesophilic habitats and emit different types of odours (earthy, aromatic and fishy). The phylogenetic results obtained by Larsson et al. [8] and Kropp et al. [12] suggest that the section Cervicolores constitutes a monophyletic group, although the phylogenetic tree generated in this study does not corroborate these conclusions, probably due to a high amount of missing data. One of the new species presented here, *Inosperma turietoense* Pancorbo & Esteve-Rav., shows a clear morphological resemblance to the Cervicolor members but is phylogenetically closer to members of the Geraniodorum group. A sequel to this work, which deals with the Cervicolor group, is currently in preparation by the authors.

In recent years, only a few new species have been described in Europe, all belonging to the Cervicolor and Maculata groups. These include *I. vinaceum* Cervini, M. Carbone & Bizio [13] in the Maculata group; another in the Cervicolor group, namely, *Inosperma monastichum* Bandini & B. Oertel [14]; *I. dodonae* Bandini & B. Oertel and *I. ismeneanum* Bandini & B. Oertel [14], which belong to the Maculata group; and, finally, *Inosperma apollonium* [15], which also belongs to the Maculatae. Based on our data, the Cervicolor group currently consists of five species in Europe. There are an additional five species that are currently being studied (Esteve-Raventós et al., in preparation). On the other hand, the Maculata group currently comprises about 12–14 species in Europe.

Among the European *Inosperma* species, *I. erubescens* A. Blytt (= *Inocybe patouillardii* Bres.) has traditionally been considered toxic due to its muscarine content [16,17]. Quite recently, several articles have pointed to the presence of various toxic substances in *Inosperma* species with tropical distribution in Asia, specifically because of their muscarine content, such as *Inosperma virosum* (C.K. Pradeep, K.B. Vrinda & Matheny) Matheny & Esteve-Rav. [18], *I. muscarium* Y.G. Fan, L.S. Deng, W.J. Yu & N.K. Zeng, *I. hainanense* Y.G. Fan, L.S. Deng, W.J. Yu & N.K. Zeng [19] and *I. zonativeliferum* Y.G. Fan, H.J. Li, F. Xu, L.S. Deng & W.J. Yu [20]. All these species belong to the two tropical evolutionary lineages mentioned above. Li et al. [21] demonstrated the presence of different neurotoxins in some species of *Inosperma* described from tropical China, although with different quantitative levels depending on the species; the presence of muscarine was detected in *I. nivallelum* S.N. Li, Y.G. Fan & Z.H. Chen (Maculata clade), and muscimol was detected in *I. longisporum* S.N. Li, Y.G. Fan & Z.H. Chen, *I. squamulosohinnuleum* S.N. Li, Y.G. Fan & Z.H. Chen and *I. squamulosobrunneum* S.N. Li, Y.G. Fan & Z.H. Chen (Cervicolores clade = sect. *Inosperma*). These results support the opinion of Stijve et al. [22], who stated that some groups of *Inocybe* are characterised by the complete absence of muscarine, including sect. *Cervicolores*.

## 2. Methods

### 2.1. Morphological Analyses

Specimens were photographed in the field with several digital cameras. After observation, fresh specimens were air-dried in a food dehydrator. Samples of specimens were rehydrated in aqueous ammonia solutions to examine anatomical features, such as cystidia, basidia and basidiospores, and observed by light microscopy. Drawings were made with the aid of a Zeiss drawing tube under an oil-immersion objective and then converted to vectors with ADOBE ILLUSTRATOR v. 25.4.1 (www.adobe.com/es/products/illustrator.html, accessed on 1 January 2024). Munsell Soil Color Charts (1994 revised edition, New York, NY, USA) were used as colour references in the macroscopic descriptions. Photographs and measurements of microscopic structures were taken with a Nikon D90 camera attached to a Nikon 55i microscope (Isaza, S.A., Nikon Spain, Barcelona, Spain) controlled with CAMERA CONTROL PRO2 v. 2.7.0 software (www.nikon.es_ES/product//apps-software/camera-control-pro-2-full-version, accessed on 1 January 2024) and subsequently retouched in ADOBE LIGHT-ROOM v. 6.14 (https://www.adobe.com/es/products/photoshop-lightroom.html, accessed on 1 January 2024) and PIXIMÈTRE v. 5.10 R1541 software (www.piximetre.fr, accessed on 1 January 2020). Measurements of microscopic structures are given as (a–)b–c(–d), where a = minimum value, b = 5% percentile, c = 95% percentile, d = maximum value, and a subscript _avg_ to indicate average values. Q-values are ratios of spore length to spore width and were calculated for each spore. The total number of spores measured (x) and the number of specimens sampled (y) are indicated as (x/y). Scanning electron micrographs (SEMs) were obtained with a Zeiss DSM-950, applying the critical point technique described in Moreno and Oltra [23]. Specimens studied have been collected in several European countries: Andorra, Finland, France, Italy, Norway, Portugal, Spain, Sweden and Switzerland. Collections were accessioned at the Herbarium of the University of Alcalá de Henares (AH) and the University of Gothenburg Herbarium (GB), with duplicates of some of them kept in the private herbaria of E. Bizio (EB), E. Larsson (EL), E. Rubio (ERD), F. Pancorbo (FP), R.J. Ferrari (FRJ), J. Ballará (JB), J. Vauras (JV) and J. Vila (JVG). Loans of specimens were obtained from the Sociedad de Ciencias Aranzadi (ARAN), the Conservatoire et Jardin Botanique de Genève (G), the University of Helsinki (H), the Naturwissenschaftlichen Sammlungen of the Tiroler Landesmuseum (IBF), the Museo di Storia Naturale di Venezia (MCVE) and the University of Turku (TUR). Herbarium codes follow Thiers [24]. Terminology follows Vellinga [25] and Kuyper [4].

### 2.2. Molecular Analyses

DNA was extracted from dried material or material stored in 1% SDS extraction buffer using the DNeasy Plant Mini Kit (Qiagen, Hilden, Germany). Old collections were DNA-extracted using the PTB DNA extraction protocol, following [26]. The following four regions were amplified: nrITS (ITS1-5.8S-ITS2), LSU, RPB1 (1200 bp; A–C) and RPB2 (5–7 region, ca. 1100 bp). The ITS (ITS1-5.8S-ITS2) and LSU regions were amplified in one piece using the primers ITS5-LR5 or separately using ITS5-ITS4 [27] for the ITS and LR0R-LR5 (or LR3) for the LSU [28]. The same primers were used for sequencing. The RPB1 region was amplified using gRPB1-A [29] and fRPB1-C rev primers [30]. The sequence spanning the RPB2 region (5–7) was amplified either in one piece, using fRPB2-5F and bRPB2-7R, or in two pieces with fRPB2-5F-gRPB2-6R and bRPB2-6F-bRPB2-7R [31,32].

PCR reactions were prepared using a Master Mix (Qiagen Multiplex PCR Kit) in a 20 μL volume, and then reactions were conducted in a GeneAmp^®^ PCR System 9700 Thermal Cycler (Applied Biosystems, San Francisco, CA, USA). The amplification programme for the ITS and LSU regions was as follows: initial denaturation at 95 °C for 5 min; then, 35–45 cycles of 95 °C for 45–60 s, 50–58 °C for 50 s and 72 °C for 1 min; followed by a final extension at 72 °C for 10 min. PCR amplifications of the RPB1 and RPB2 regions followed O’Donnell et al. [33] and Hansen et al. [34], respectively. PCR products were visualised in a 1% agarose gel and stained with SYBR Safe DNA Gel Stain (Invitrogen-Thermo Fisher Scientific, Inc., Waltham, MA, USA), using a UV trans-illuminator. PCR products were purified using Exo-sap-IT (USB Corporation, Santa Clara, CA, USA) and then sequenced at the Molecular Biology Service of Alcalá University. The generation of ITS and LSU sequence data at the University of Gothenburg followed the methods described in Nitare et al. [35].

Sequences were edited and assembled in SEQUENCHER v. 4.10.1 (Gene Codes Corp., Ann Arbor, MI, USA) and deposited in GenBank (Table 1). Additional sequences were downloaded from GenBank. Nucleotide sequences of each region were automatically aligned in ALIVIEW [36] and then manually adjusted. Protein-coding genes were translated to amino acids to determine intron positions. To check gene congruence, each region was analysed in IQ-TREE, starting from a random tree under default options. To assess the branch confidence, 1000 ML bootstrap replicates were conducted using ultrafast bootstrapping. Gene congruence was assessed by comparing supported clades among single-gene genealogies [37]. Clades were considered in conflict if a supported clade (ML-BP ≥ 95%) for one marker was contradicted with significant support by another one. Since no conflicts were detected, the four markers were concatenated. Introns were included, and the third codon position was partitioned in the RPB1 and RPB2 regions. Ten partitions were set: ITS1, 5.8S, ITS2, LSU, RPB1 (1st and 2nd positions), RPB1 (3rd position), RPB1 introns, RPB2 (1st and 2nd positions), RPB2 (3rd position) and RPB2 introns.

Maximum likelihood (ML) analysis was carried out in IQ-TREE [38], starting from a random tree under default options. To assess the branch confidence, 1000 ML bootstrap replicates were conducted using standard bootstrapping. Bayesian analysis was conducted in MRBAYES v. 3.2.7a [39], in the CIPRES Science Gateway [40], using two parallel runs of 4 Metropolis-coupled Markov chain Monte Carlo (MCMCMC) chains for 20 M generations, starting from a random tree, and sampling one tree every 100 generations from the posterior distribution. Substitution models were sampled across the GTR space during the MCMC simulation [39]. Stationarity was assumed when the average standard deviation of split frequencies fell below 0.01. The burn-in fraction was set to discard 0.25 trees from each analysis. To assess the branch confidence, a 50% majority rule consensus tree was computed with the remaining trees using the SUMT command of MRBAYES. An ML standard bootstrap (ML-Boot) ≥ 70 or Bayesian posterior probability (PP) values ≥ 0.95 were considered supported.

**Table 1:** List of collections used in the molecular analyses. The sequences generated in this study are in bold.

Species	Country	Putative Host	Voucher	GenBank Accession No.			Reference
				ITS	LSU	RPB1/2	
***Inosperma subhirsutum***	**Norway**	***Betula nana*, *Salix reticulata***	**EL 76-12 (GB 0243034)**	**OR817729**	**OR817729**	**PP092163**	**This study**
*Inosperma subhirsutum* (as *I.calamistrata*)	Latvia	—	JV 11950 (WTU, TUR-A)	EU555452	EU555452	AY333763	[41]
***Inosperma subhirsutum***	**Finland**	***Populus tremula*, *Betula pubescens*, *Alnus incana***	**EL 352-13 (GB 0243043)**	**OR817728**	**OR817728**	**—**	**This study**
***Inosperma subhirsutum***	**Sweden**	***Salix reticulata***	**EL 198-18 (GB 0243041)**	**OR817730**	**OR817730**	**PP092164**	**This study**
***Inosperma subhirsutum***	**Sweden**	***Betula pubescens*, *Salix* spp.**	**EL 142-14 (GB 0243040)**	**OR817727**	**OR817727**	**PP092177/** **PP092162**	**This study**
***Inosperma subhirsutum***	**Italy**	***Salix herbacea***	**EB 1992082601 (dupl. AH 56195)**	**PP431507**	**PP431530**	**—**	**This study**
***Inosperma subhirsutum* (as *Inocybe subhirsuta*)**	**France**	***Salix reticulata***	**R. Kühner 72-102 (G 00052221) Holotype**	**PP065739**	**—**	**—**	**This study**
***Inosperma subhirsutum***	**Sweden**	***Salix herbacea***	**AH 46825**	**PP431508**	**—**	**—**	**This study**
*Inosperma subhirsutum* (as *Inocybe calamistrata*)	Norway	*Dryas octopetala*, *Salix reticulata*	EL 43-05 (GB 0248040)	AM882947	AM882947	—	[42]
*Inosperma subhirsutum* (as *Inocybe calamistrata*)	Norway	*Salix herbacea*	EL 26-05 (GB 0248023)	AM882946 (ITS2)	AM882946	PP092179/PP092165	[42]
***Inosperma subhirsutum***	**Italy**	***Salix* sp., *Picea abies***	**EB 2014080711 (dupl. AH 56193)**	**PP431509**	**PP431531**	**—**	**This study**
*Inosperma neohirsutum* (as *Inocybe calamistrata*)	Norway	*Quercus*, *Corylus*	NOBAS 1849-16	UDB07673483	—	—	UNITE database
***Inosperma neohirsutum***	**Sweden**	***Pinus sylvestris*, *Betula pendula***	**EL 163-15 (GB 0207661)**	**OR831119**	**OR831119**	**PP092169**	**This study**
*Inosperma neohirsutum* (as *Inocybe* cf. *calamistrata*)	Sweden	Mixed coniferous forest	EL 77-03 (GB 0150442)	AM882945	AM882945	—	[42]
***Inosperma neohirsutum***	**Spain**	***Fagus sylvatica***	**AH 26947 Holotype**	**PP431510**	**PP431532**	**—**	**This study**
***Inosperma neohirsutum***	**Spain**	***Fagus sylvatica***	**AH 24593**	**PP431511**	**—**	**—**	**This study**
***Inosperma neohirsutum***	**France**	***Fagus sylvatica***	**AH 48235**	**PP431512**	**PP431533**	**—**	**This study**
*Inosperma apiosmotum*	United States	Mixed forest (*Fagus*, *Tsuga*, *Pinus*, *Quercus*)	PBM 2784 (TENN 062582)	JQ801384	EU555454	EU555453	GenBank (unpubl.)
*Inosperma apiosmotum*	Canada	In gravel and humus in a swamp	WTU (F 043329) Isotype	NR_121487	—	—	GenBank (unpubl.)
*Inosperma apiosmotum*	United States	Coniferous forest (*Tsuga*, *Pinus*, *Picea*)	PBM 3020 (TENN 062779)	JQ801385	JN975021	JQ846463	GenBank (unpubl.)
*Inocybe* sp. (as *Inocybe* aff. *calamistrata*)	United States	Coniferous forest (*Abies*, *Tsuga*)	SAT 9826004	JQ801387	JQ815410	MK415439/JQ846467	GenBank (unpubl.)
*Inocybe* sp. (as *Inocybe calamistrata*)	United States	—	PBM 2351(WTU)	—	AY380368	AY351794/AY333764	[32]
*Inosperma maximum* (as *Inocybe hirsuta* var. *maxima*)	United States	In coniferous duff	MTS 2732 (UC)	JQ801400	JQ815420	—	[12]
*Inosperma maximum*	United States	*Tsuga*, *Pseudotsuga*	PBM 2222 (WTU)	—	EU569854	—	[12]
*Inosperma mutatum*	United States	*Tsuga*	PBM 2953 (TENN 062711)	JQ801410	JQ994476	JQ846488	[12]
*Inosperma mutatum* (as *Inocybe mutata*)	United States	—	PBM 2542 (CUW)	—	AY732212	DQ447917/DQ472729	GenBank (unpubl.) –LSU- [1] –RPB1, RPB2-
*Inosperma latericium*	New Zealand	*Nothofagus menziesii*	ZT 8481 (PDD 71261)	JQ801402	JN975024	—	[7,12]
*Inosperma calamistratoides*	New Zealand	*Nothofagus*	ZT 67262 (PDD 27121) Holotype	KJ756494	—	—	[7]
*Inosperma calamistratoides*	New Zealand	*Nothofagus*	PBM 3112 (TENN, PDD 97855)	—	JQ815414	JQ846473	[7,12]
*Inosperma calamistratoides*	New Zealand	*Nothofagus*	ZT 9630 (PDD 72711)	JQ801392	AY380369	AY351795/AY333765	[7,12]
*Inosperma calamistratoides*	Tasmania	*Nothofagus cunninghamii*, *Leptospermum*	PBM 3384 (TENN 065750)	JQ801393	JQ815415	KJ729949	[7,12]
*Inosperma cyanotrichium*	Tasmania	*Eucalyptus*	PERTH 08516952	JQ801397	JN975033	JQ846476	[7]
*Inosperma viridipes*	Australia	*Eucalyptus*	PBM 3767 (PERTH 0836390) Holotype	KP641645	KP171094	—	[7]
*Inosperma longisporum*	China	*Abies georgei*	MHHNU 32237	OP135509	OP133999	OP161560	[21]
*Inosperma longisporum*	China	*Abies georgei*	MHHNU 33070	OP135504	OP135495	OP161564	[21]
***Inosperma calamistratum***	**Spain**	***Castanea sativa***	**AH 36309**	**PP431513**	**PP431534**	**PP478160/PP478207**	**This study**
***Inosperma calamistratum***	**Portugal**	**Mixed forest**	**AH 46636**	**PP431514**	**PP431535**	**PP478161**	**This study**
***Inosperma calamistratum***	**Spain**	***Alnus glutinosa***	**AH 27000**	**PP431515**	**—**	**—**	**This study**
***Inosperma calamistratum***	**Sweden**	**Deciduous forest**	**IBF 19790433 Neotype**	**PP431543 (ITS1)/PP431547 (ITS2)**	**—**	**—**	**This study**
***Inosperma calamistratum***	**Spain**	***Larix decidua***	**ARAN 00235**	**PP431516**	**PP431536**	**—**	**This study**
*Inosperma calamistratum* (as *Inocybe calamistrata*)	Sweden	*Pinus sylvestris*, *Betula pendula*	EL 19-04 (GB 240829)	AM882938	AM882938	—	[42]
***Inosperma calamistratum***	**Spain**	***Quercus suber***	**AH 56397**	**PP431517**	**PP431537**	**—**	**This study**
***Inosperma calamistratum***	**Sweden**	**Mixed coniferous forest**	**JO 120916 (GB 0181745)**	**OR803781**	**OR803781**	**PP092166**	**This study**
***Inosperma calamistratum***	**France**	***Pinus nigra* subsp. *laricio***	**EL 362-19 (GB 0237702)**	**OR803784**	**OR803784**	**—**	**This study**
***Inosperma calamistratum***	**Spain**	***Pinus sylvestris***	**AH 40200**	**PP431518**	**PP431538**	**PP478162**	**This study**
***Inosperma calamistratum***	**Sweden**	**Mixed forest**	**EL 446-17 (GB 0237701)**	**OR803783**	**OR803783**	**PP092178/PP192167**	**This study**
***Inosperma gracilentum***	**Sweden**	***Dryas octopetala*, *Salix reticulata*, *Bistorta vivipara***	**EL 85-19 (GB 0207620) Holotype**	**OR817726**	**OR817726**	**—**	**This study**
***Inosperma gracilentum***	**Switzerland**	***Salix retusa***	**J. Favre Z.A.82a (G 00551725)**	**PP431544 (ITS1)/PP431548 (ITS2)**	**—**	**—**	**This study**
*Inocybe* sp. (as *Inosperma calamistratum*)	Canada	—	UBC-F 19681	HM240530	HM240530	—	GenBank (unpubl.) [12] as *I. atrovirescens* (prov.)
*Inocybe* sp. (as *Inocybe hirsuta* var. *maxima*)	United States	—	PBM 1066 (WTU)	—	AY038317	AF389539/AY333766	[30]
*Inosperma veliferum* (as *Inocybe geraniodora*)	Italy	*Salix retusa*, *S. reticulata*, *Dryas octopetala*	MCVE 4485	JF908117	—	—	[43]
***Inosperma veliferum***	**Italy**	***Salix retusa*, *S. reticulata***	**EB 1993080906 (dupl. AH 56198 and MCVE 20884)**	**PP431519**	**PP431539**	**—**	**This study**
***Inosperma veliferum* (as *Inocybe geraniodora* var. *velifera*)**	**France**	***Salix herbacea***	**R. Kühner 71-143 (G 00110853) Holotype**	**PP431520**	**—**	**—**	**This study**
***Inosperma veliferum***	**Italy**	***Salix retusa*, *S. reticulata*, *Dryas octopetala*, *Bistorta vivipara*, *Kalmia procumbens***	**AH 46962**	**PP431521**	**—**	**—**	**This study**
***Inosperma veliferum***	**Spain**	***Salix reticulata*, *Dryas octopetala***	**AH 21346**	**PP431522**	**—**	**—**	**This study**
*Inosperma geminum* (as *Inocybe geraniodora*)	Sweden	*Dryas octopetala*	EL 106-06 (GB 0207617)	FN550945	FN550945	—	[44]
***Inosperma geminum***	**Sweden**	***Dryas octopetala***	**EL 63-06 (GB 0207619)**	**OR823936**	**OR823936**	**PP092172**	**This study**
***Inosperma geminum***	**Sweden**	***Dryas octopetala*, *Salix reticulata***	**GB 0207616**	**OR823939**	**OR823939**	**—**	**This study**
***Inosperma geminum***	**Sweden**	***Dryas octopetala***	**EL 50-17 (GB 0207618)**	**OR823938**	**OR823938**	**PP092173**	**This study**
***Inosperma geminum***	**Sweden**	***Dryas octopetala*, *Salix reticulata*, *S. herbacea***	**JV 31497 (TUR) Holotype**	**OR823936**	**OR823936**	**PP092174**	**This study**
***Inosperma geraniodorum***	**Italy**	***Dryas octopetala***	**EB 2008081002 (dupl. AH 56199)**	**PP431523**	**—**	**—**	**This study**
***Inosperma geraniodorum***	**Sweden**	***Dryas octopetala*, *Salix reticulata***	**EL 156-17 (GB 0243140)**	**OR823942**	**OR823942**	**PP092176**	**This study**
***Inosperma geraniodorum***	**Sweden**	***Dryas octopetala*, *Salix reticulata***	**EL 126-16 (GB 0243139)**	**OR823943**	**OR823943**	**PP092175**	**This study**
***Inosperma geraniodorum***	**Italy**	***Salix retusa*, *Dryas octopetala***	**EB 2019080304 (dupl. AH 46961)**	**PP431524**	**—**	**—**	**This study**
***Inosperma geraniodorum***	**Switzerland**	***Alnus alnobetula***	**J. Favre Z.A.82b (G 00052203) Lectotype**	**PP431545 (ITS1)/PP431549 (ITS2)**	**—**	**—**	**This study**
***Inosperma geraniodorum***	**Norway**	***Salix reticulata***	**EL 105-12 (GB 0243141)**	**OR823941**	**OR823941**	**—**	**This study**
***Inosperma turietoense***	**Spain**	***Abies alba*, *Fagus sylvatica***	**AH 47669**	**PP431525**	**PP431540**	**—**	**This study**
***Inosperma turietoense***	**Spain**	***Abies alba*, *Fagus sylvatica***	**AH 47710 Holotype**	**PP431526**	**PP431541**	**PP478208**	**This study**
*Inosperma latericium*	New Zealand	*Nothofagus fusca*, *N. menziesii*	PDD 27120 Type	KP171150	—	—	[7]
***Inosperma praetermissum***	**Sweden**	***Picea abies*, *Corylus avellana***	**EL 4-14 (GB 0243035)**	**OR831122**	**OR831122**	**—**	**This study**
*Inosperma praetermissum* (as *Inosperma calamistratum*)	United States	*Abies*, *Tsuga*, *Picea*	PBM 1105 (WTU)	JQ801386	JQ815409	MK415438/JQ846466	[12]
*Inosperma praetermissum* (as *Inocybe calamistrata*)	Sweden	*Betula pendula*, *Corylus avellana*	EL 130-04 (GB 0240811)	AM882944	AM882944	—	[42]
***Inosperma praetermissum***	**Italy**	***Picea abies***	**EB 2019081705 (dupl. AH 46960)**	**PP431527**	**—**	**—**	**This study**
***Inosperma praetermissum***	**Italy**	**Alpine scrubland**	**EB 2019083003 (dupl. AH 46963)**	**PP431528**	**—**	**—**	**This study**
***Inosperma praetermissum***	**Andorra**	***Salix herbacea***	**AH 46901 Isotype of *I. geraniodora* var. *gracilenta* f. *salicis-herbaceae*)**	**PP431546 (ITS1)/PP431550 (ITS2)**	**—**	**—**	**This study**
***Inosperma praetermissum***	**Sweden**	***Salix herbacea*, *Bistorta vivipara***	**EL 206-13 (GB 0243036)**	**OR831121**	**OR831121**	**PP092180/PP092171**	**This study**
***Inosperma praetermissum***	**Sweden**	***Salix herbacea*, *Bistorta vivipara***	**EL 130-19 (GB 0243038)**	**OR831124**	**OR831124**	**—**	**This study**
***Inosperma praetermissum***	**Sweden**	***Salix* spp., *Betula nana***	**EL 161-18 (GB 0243037)**	**OR831120**	**OR831120**	**PP092170**	**This study**
***Inosperma praetermissum (as Inocybe praetermissa)***	**Finland**	***Pinus***	**P.A. Karsten 2497 (H) Holotype**	**PP431551 (ITS2)**	**—**	**—**	**This study**
***Inosperma cervicolor***	**Sweden**	***Quercus*, *Tilia*, *Corylus***	**EL 101-14**	**PP431529**	**PP431542**	**PP478159/PP478206**	**This study**
*Mallocybe tomentella*	United States	*Quercus montana*, *Juniperus*	PBM 4138 (TENN 071837)	MG773814	MK421969	MK415443/MH577506	GenBank (unpubl.)
*Mallocybe terrigena*	Sweden	*Picea abies*	EL 117-04	AM882864	AY380401	AY333301/AY333309	[42]
*Auritella fulvella*	Australia	*Corymbia citriodora*, *Eucalyptus crebra*	BRI:AQ 669485 (BRI)	KJ702355	KJ702352	MK415422/KJ702357	[7]

### 2.3. Molecular Results

A total of 111 (45 ITS1+2, 4 ITS1, 5 ITS2, 34 LSU, 7 RPB1 and 16 RPB2) sequences were generated for this study (Table 1). In addition, 78 sequences from GenBank and 1 from UNITE were used in the analyses. The concatenated alignment contained 85 taxa and 3388 unambiguously aligned nucleotide positions (405 ITS, 540 LSU, 1281 RPB1 and 1162 RPB2). The missing data percentage was 60%.

**Figure 1:**
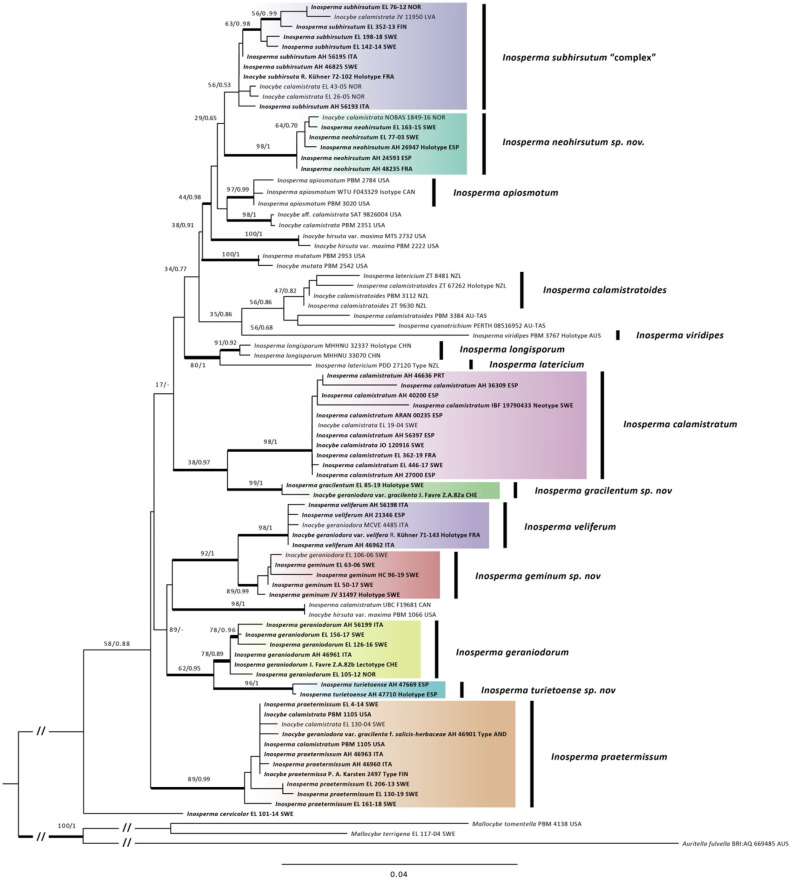
Most-probable ML tree of the ITS, LSU, RPB1 and RPB2 sequences of the *Inosperma calamistratum* and *I. geraniodorum* groups. Bootstrap ML values/posterior probabilities from Bayesian analysis are shown around the branches. Thick branches indicate nodes with phylogenetic support in at least one of the analyses (bootstrap values ≥ 70% and posterior probability ≥ 0.95). Sequences of *Mallocybe tomentella*, *M. terrigena* and *Auritella fulvella* were used to root the tree. The country of origin of each collection is abbreviated by ISO Alpha-3 codes. Specimens sequenced in this work are marked in bold.

The ML analysis of the combined dataset resulted in a single best ML tree of −lnL = 12,847,950. The Bayesian analysis reached average standard deviations of split frequencies > 0.01 after 12,195,000 generations. The Bayesian majority rule consensus tree is shown in Figure 1, with bootstrap support values and Bayesian posterior probabilities by nodes. The backbone of the tree is overall not supported, but shallow clades corresponding to *I. neohirsutum* (ML-Boot 98, BPP 1), *I. calamistratum* (ML-Boot 98, BPP 1), *I. gracilentum* (ML-Boot 99, BPP 1), *I. veliferum* (ML-Boot 98, BPP 1), *I. geraniodorum* (ML-Boot 78, BPP 0.89), *I. turietoense* (ML-Boot 96, BPP 1) and *I. praetermissum* (ML-Boot 89, BPP 0.99) received support in at least one analysis among European taxa. The clade encompassing samples assigned to *I. subhirsutum* showed a rather high sequence divergence and was not supported (ML-Boot 61, BPP 0.53), but a smaller clade within it containing five samples from Northern Europe was supported in the Bayesian analysis (ML-Boot 63, BPP 0.98). A larger clade comprising sequences of *I. subhirsutum* and *I. neohirsutum* and sequences from North America identified as *I. apiosmotum*, *I. hirsutum* var. *maximum* and *I. calamistratum* was also supported in the Bayesian analysis (ML-Boot 44, BPP 0.98).

## 3. Taxonomy

(I) Calamistratum Group

1. *Inosperma calamistratum* (Fr.) Matheny & Esteve-Rav.;
2. *Inosperma gracilentum* E. Larss. & Esteve-Rav., sp. nov.;
3. *Inosperma neohirsutum* Esteve-Rav., Pancorbo & E. Larss., sp. nov.;
4. *Inosperma praetermissum* (P. Karst.) Esteve-Rav., E. Larss. & Pancorbo comb. nov.;
5. *Inosperma subhirsutum* (Kühner) Matheny & Esteve-Rav.

(II) Geraniodorum Group

6. *Inosperma geraniodorum* (J. Favre) Matheny & Esteve-Rav.;
7. *Inosperma geminum* E. Larss. & Vauras, sp. nov.;
8. *Inosperma turietoense* Pancorbo & Esteve-Rav., sp. nov.;
9. *Inosperma veliferum* (Kühner) Matheny & Esteve-Rav.

***Inosperma calamistratum* (Fr.) Matheny & Esteve-Rav., Mycologia 112(1): 94, 2019.**

MycoBank No. 830345

**Figure 2:**
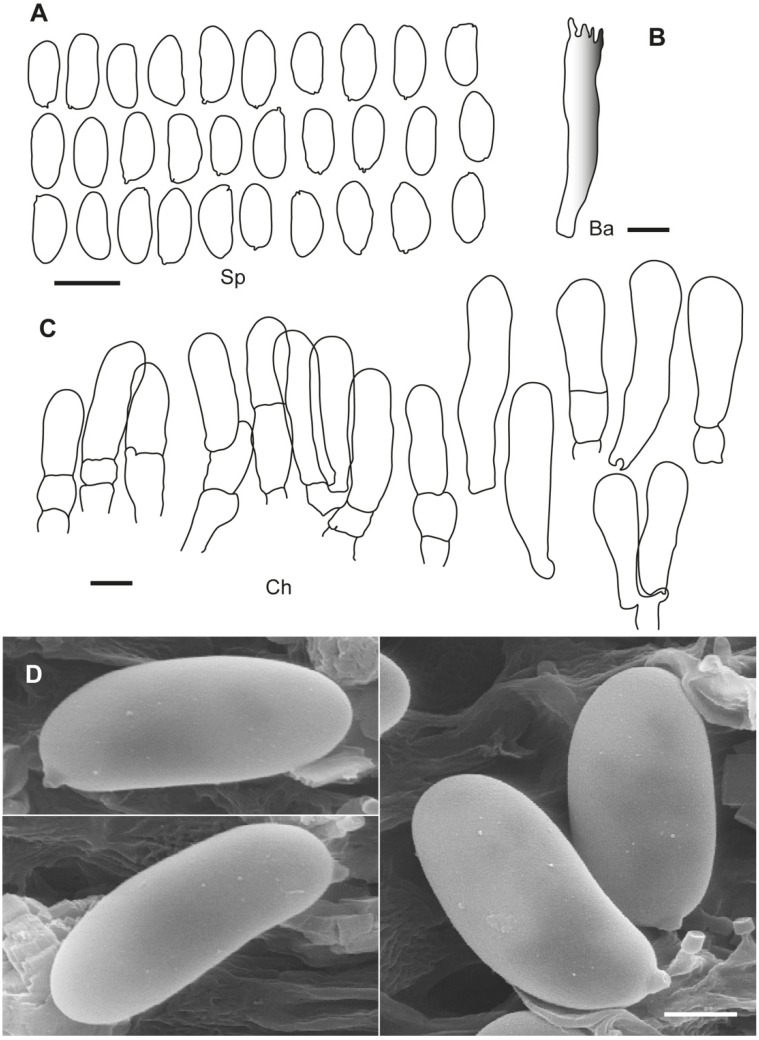
*Inosperma calamistratum* Neotype IBF 19790433. (**A**) Basidiospores. (**B**) Basidia. (**C**) Cheilocystidia. (**D**) Spore SEMs. Scale bars: 10 µm (**A**–**C**); 2 µm (**D**).

Figure 2 and Figure 9A–C

≡ *Agaricus calamistratus* Fr., Systema Mycologicum 1: 256, 1821, nom. sanct. MycoBank No. 239098.

≡ *Inocybe calamistrata* (Fr.) Gillet, Les Hyménomycètes ou Description de tous les Champignons qui Croissent en France 1: 513, 1876. MycoBank No. 232510.

= *Agaricus hirsutus* Lasch, Linnaea 4: 546, 1829, nom. sanct. Systema Mycologicum 3 (Index): 23, 1832. MycoBank No. 461481.

= *Inocybe hirsuta* (Lasch: Fr.) Quél., Mémoires de la Société d’Emulation de Montbéliard 2, 5: 178, 1872. MycoBank No. 191743.

= *Inosperma hirsutum* (Lasch: Fr.) Matheny & Esteve-Rav., Mycologia 112(1): 103, 2019. MycoBank No. 831768.

**Neotype.** Sweden, Småland, Femsjö: between moss and grass, not far from the edge of a deciduous forest, 135–170 m alt., 1 September 1979, leg. M. Moser, IBF 19790433, GenBank accession: ITS1 (PP431543), ITS2 (PP431547). Neotype designated by T.W. Kuyper ([4]: 36). MBT 10016187.

**Descriptions and selected iconography.** Konrad and Maublanc ([45], pl 89, 90), Bresadola ([46], pl 720 Figure 1), Heim ([47], pl 3 Figure 2), Kühner ([48]: 200–201)**,** Phillips ([49]: 148), Moser and Jülich ([50], pl 9 Figure 1), Kuyper ([4]: 35–36, pro parte), Bon ([51]: 235), Leisner and Kalamees ([52]: 102, pl 4), Stangl ([53]: 54–57, pl 3), Nespiak ([54]: 20–22), Courtecuisse and Duhem ([55], n° 1022), Bon ([5]: 26, pro parte), Courtecuisse ([56]: 152, 500), Breitenbach and Kränzlin ([57]: 46–47 n° 8, pro parte), Dähncke ([58]: 646), Ferrari ([59]: 54–56, 328 top), Roux ([60]: 772), Eyssartier and Roux ([61]: 872), Outen and Cullington ([62]: 16), Ludwig ([63]: 203–204, pl 129.18 C-D, pro parte), Laessoe and Petersen ([64]: 658).

**Additional microscopic examination of the neotype of *I. calamistrata*.** *Basidiospores* (9.0–)9.5–11.5(–11.8) × (4.5–)4.6–5.6(–5.7) µm, Sp_avg_ = 10.4 × 5.1 µm, Q = (1.8–)1.83–2.3(–2.6), Q_avg_ = 2.0 (n = 30), smooth, subcylindrical to narrowly ellipsoid in face view, phaseoliform to subphaseoliform in profile, pale greenish glaucous. *Basidia* clavate, mainly four-spored, 36–40 × 7.5–9.5 µm, with evenly distributed intracellular greenish pigment. *Pleurocystidia* absent. *Lamellar trama* pale, hardly pigmented, except for cells with greenish content. *Lamellar edge* sterile, uniform white. *Cheilocystidia* tightly packed, subcylindrical to narrowly clavate, (22.1–)26.2–40.1 × 7.2–9.7(–10.2) µm, Ch_avg_ = 31.3 × 8.3 µm (n = 16), subcylindrical to subclavate or clavate, with rounded apex, usually two- to three-septate at base, hyaline, thin-walled. *Clamp connections* present.

**Distribution**. Based on the data obtained in our study, *I. calamistratum* is widespread throughout Europe, as also suggested by Courtecuisse and Duhem [55], although it is not as common as it has been considered until now. The high number of observations and records of this species may be due to its apparent ease of recognition, based on its colouration and the appearance of the surfaces of the pileus and stipe. Most records reported from the northern boreal to alpine zones do not correspond to *I. calamistratum* but probably refer to *I. praetermissum*, *I. gracilentum* and *I. subhirsutum*. Furthermore, records from similar bioclimatic areas may correspond to *I. neohirsutum*, with which it is more easily confused.

There are no records in the GenBank and UNITE databases referring to other continents, such as North America and Asia (Matheny, pers. comm.). The only record matching the sequence obtained from the neotype corresponds to AM882938 (EL 19-04, GB 0240829), from Sweden. The deposited sequences matching other similar species, especially *I. praetermissum* and *I. subhirsutum*, are more frequent.

**Ecology.** *Inosperma calamistratum* occurs in Europe in humid to very moist habitats on poor to rich soils, usually acidic, in both continental and Atlantic climates, in nemoral to montane forests, often along paths. In Sweden, where the species was described, it occurs from the nemoral to boreo-nemoral zone, usually found in mixed conifer-dominated forests of *Vaccinium uliginosum* type, on granitic bedrock ground, and associated with *Betula pendula*, *Picea abies* and *Pinus sylvestris.* We are not aware of any records from the northern boreal to the alpine zone, where other species occur. In Europe, it is associated and likely forms ectomycorrhizae with both broadleaved trees (*Betula*, *Alnus*, *Quercus* and *Fagus*) and conifers (*Pinus*, *Picea*, *Larix* and *Pseudotsuga*) in natural and reforested woodlands (e.g., AH 46636 and ARAN 00235).

**Etymology.** Derived from the Latin *calamister*, meaning crisp or curled, in reference to the scaly hirsute or squarrose surface of the pileus and stipe.

**Additional specimens examined.** France, Corsica, Haute-Corse, Corte, Restonica Valley: 42°15′7.44″ N, 9° 3′39.09″ E, in *Pinus nigra* subsp. *laricio* forest in acidic soil, 1130 m alt., 8 November 2019, leg. P.A. Moreau, N. Subervielle & E. Larsson, EL 362-19 (GB 0237702), GenBank accession: ITS-LSU (OR803784). Portugal, Viseu, Moselos: 40°40′55.68″ N, 7°57′29.54″ W, mixed partially reforested forest with *Pinus pinaster*, *Pseudotsuga menziesii*, *Acacia melanoxylon*, *Eucalyptus* sp. and *Quercus robur*, in acidic soil, 540 m alt., 7 November 1996, leg. F. Esteve-Raventós, AH 46636, GenBank accession: ITS (PP431514), LSU (PP431535), RPB1 (PP478161). Guarda, Serra do Estrela, Manteigas: 40°25′15″ N, 7°35′23″ W, under *Betula alba* in acidic soil, 1295 m alt., 10 Nov. 2015, leg. M.A. Ribes, J.F. Mateo, M. Parreño & F. Pancorbo, AH 51034 (dupl. FP 15111006). Spain, Aragón, Valle de Hecho, Selva de Oza: 42°50′6″ N, 0°42′33″ W, under *Abies alba* and *Fagus sylvatica* in acidic soil, 1150 m alt., 17 September 2023, leg. F. Pancorbo & F. Esteve-Raventós, AH 58564. Asturias, Allande, San Emiliano: 43°15′53.13″ N, 6°49′33.76″ W, in *Quercus suber* forest, in acidic soil, 260–270 m alt., 22 December 2020, leg. I. Martín, AH 56397 (dupl. ERD 8644), GenBank accession: ITS (PP431517), LSU (PP431537). Castilla-La Mancha, Guadalajara, Peñalba de la Sierra, ribera del arroyo de Cañamar: 41° 8′43.02″ N, 3°23′23.73″ W, under *Alnus glutinosa* and *Quercus pyrenaica* nearby, in acidic soils, 1290 m alt., 14 July 2002, leg. J.P. Campos & J.C. Campos, AH 29995. Castilla y León, Segovia, Valsaín-Puerto de Navacerrada: 40°49′22.86″ N, 4°0′49.90″ W, in *Pinus sylvestris* forest in acidic soil, 1360 m alt., 22 November 2008, leg. A. Sánchez, AH 40200, GenBank accession: ITS (PP431518), LSU (PP431538), RPB1 (PP478162). Ibidem: 10 October 2015, leg. A. Sánchez, AH 46926. Castilla y León, Zamora, Galende, bank of river Tera: 42°6′55.18″ N, 6°41′10.42″ W, under *Alnus glutinosa* in acidic soil, 1000 m alt., 19 October 1999, leg. M. Castro-Cerceda, AH 27000, GenBank accession: ITS (PP431515). Galicia, Lugo, Cervantes, Vilarnovo: 42°53′37.11″ N, 6°58′38.87″ W, in humid *Castanea sativa* forest, in acidic soil, 660 m alt., 4 October 1994, leg. F. Esteve-Raventós, AH 36309, GenBank accession: ITS (PP431513), LSU (PP431534), RPB1 (PP478160), RPB2 (PP478207). Madrid (Community), Rascafría: 40°51′32.67″ N, 3°54′39.53″ W, in boggy soil in *Pinus sylvestris* forest with *Betula alba* and *Salix atrocinerea*, in acidic soil, 1278 m alt., 22 August 2013, leg. F. Pancorbo, AH 44420 (dupl. FP 13082210). Madrid (Community), Cercedilla: under *Pinus sylvestris* in acidic soil, 22 November 2021, leg. P. Miranda, AH 49307. Navarra (Nafarroa), Areso-Labaki: 43°5′29.42″ N, 1°57′3.52″ W, in a *Larix decidua* reforested forest, 530 m alt., 30 August 2014, leg. P. Arrillaga, ARAN 00235, GenBank accession: ITS (PP431516), LSU (PP431536). País Vasco (Euskadi), Guipúzcoa (Gipuzkoa), Irún, Peñas de Aia (Aiako Harria): in reforested *Larix decidua* forest, 350–400 m alt., 18 November 1991, leg. J.M. Lekuona, AH 22169. Sweden, Bohuslän, Resteröd, Ulvesund: along path in mixed coniferous forest under *Pinus sylvestris* and *Betula pendula* on acidic soil, 25 July 2004, leg. E. Larsson, EL 19-04 (GB 0240829), GenBank accession: ITS-LSU (AM882938)—as *Inocybe calamistrata*. Västergötland, Vänersborg, Toltorp: in mixed coniferous forest on acid soil, 16 September 2016, leg. J. Olsson, JO 120916 (GB 0181745), GenBank accession: ITS-LSU (OR803781), RPB2 (PP092166). Västergötland, Trollhättan, Jonstorp: in pasture with conifer and deciduous trees, 14 October 2017, leg. J. Olsson, EL 446-17 (GB 0237701), GenBank accession: ITS-LSU (OR803783), RPB1 (PP092178), RPB2 (PP092167). Västergötland, Sandhult, Sandhults hembyggdgård: in a pasture close to *Pinus sylvestris*, *Betula pendula* and *Quercus robur* on acidic soil, 17 September 2013, leg. E. Larsson, EL 404-13 (GB 0237700), GenBank accession: ITS-LSU (OR803782).

**Notes**. *Inosperma calamistratum* (≡ *Agaricus calamistratus* Fr.) is the type of the genus *Inosperma* (Kühner) Matheny & Esteve-Rav. [2]. Because of its morphological and ecological peculiarities, it is easy to recognise and has been frequently recorded in Europe. The surface of the pileus and stipe is characteristically scaly–hirsute, even squarrose (Figure 9A–C). The blue-green colour at the base of the stipe is also very distinctive, although this character can vary with environmental conditions, age and degree of imbibition of the basidiomata. Microscopically, the narrow spores show a marked tendency to be phaseoliform in profile (Figure 2A,D), and the lamellar edge consists of numerous claviform to subcylindrical, two- to three-septate cheilocystidia, barely longer than 40 µm (Figure 2C), mixed with some basidia, often greenish pigmented. Its odour is very peculiar and variable, and it has been defined in different ways in the literature, such as rancid, resinous, sour, reminiscent of fish or with a sweet fruity component. In any case, it is not like that of pelargonium, which is also present in other related species, especially those defined here as the Geraniodorum group. Based on the results of our study, we are convinced that *I. calamistratum* has often been confused with other species, and its presence in northern boreal to alpine zones seems most likely to be excluded in view of the results obtained. Records from high altitudes and boreo-alpine latitudes correspond to other morphologically very close species that have been identified in the past as ecological forms or variants of *I. calamistratum* ([65]: 77).

On the European continent, *I. calamistratum* shares similar or common habitats with *I. neohirsutum*, and both occur in moist temperate forests of *Fagaceae* (*Fagus*, *Quercus* and *Castanea*), coniferous or mixed. Morphologically they are also similar in appearance, although *I. calamistratum* often produces larger basidiomata, with a longer, slender and elastic stipe (30–80 × 2–8 mm), the scales of the pileus and stipe are thinner and often recurved, giving it a hirsute to squarrose appearance, and are usually distributed over the entire surface as they develop. In *I. neohirsutum*, the scales are conspicuously aggregated on the central part of the pileus during development, then appear thicker as they tend to fuse and take on a pyramidal appearance. There are also differences in spore Q, and although both have a clear tendency to be phaseoliform in profile, *I. neohirsutum* has slightly wider spores with a lower Q (Q_avg_ 1.7 vs. 2.0). Other species of similar appearance, such as *I. gracilentum*, *I. praetermissum* and *I*. *subhirsutum*, show differences in spore shape and size, inhabit different ecosystems and are well-separated phylogenetically (Figure 1).

There are several morphologically similar species in North America, but they are all distinct phylogenetically [12,66]. *Inosperma mucidiolens* (Grund & D.E. Stuntz) Matheny & Esteve-Rav. (= *Inocybe calamistrata* var. *mucidiolens* Grund & D.E. Stuntz) produces a characteristic odour of green corn, while the basidiomata of *Inosperma apiosmotum* (Grund & D.E. Stuntz) Matheny & Esteve-Rav. smell particularly of ripe pears. Other species, such as *Inosperma maximum* (A.H. Sm.) Matheny & Esteve-Rav., are characterised by their large, robust size and long stipe (55–120 × 2.5–6 mm).

Also similar in appearance to *I. calamistratum* are the species *Inosperma longisporum*, *I. squamulosobrunneum* and *I. squamulosohinnuleum*. These have been recently described from China, from montane coniferous forests in subtropical environments. All three species show macroscopic and microscopic differences from *I. calamistratum* and phylogenetic characters closer to North American species than to European species [21]. Other similar species, especially in pileus and stipe cover, have been recorded from the Australian continent and Southeast Asia, e.g., *Inosperma calamistratoides* (E. Horak) Matheny & Esteve-Rav. and *Inosperma latericium* (E. Horak) Matheny & Esteve-Rav., but with different micromorphological characters and rather distant phylogenetically [7,67,68].

***Inosperma gracilentum* E. Larss. & Esteve-Rav., sp. nov.**

MycoBank No. 850652

**Figure 3:**
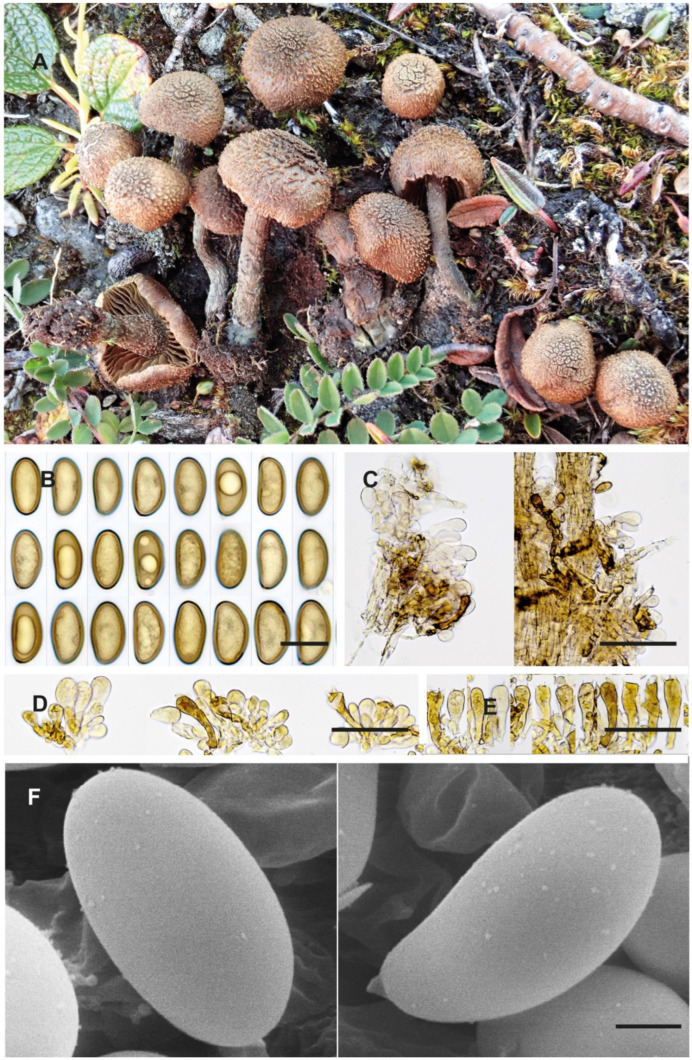
*Inosperma gracilentum* Holotype EL 85-19 (GB 0207620). (**A**) Basidiomata. (**B**) Basidiospores. (**C**) Caulocystidia. (**D**) Cheilocystidia. (**E**) Basidia. (**F**) Spore SEMs. Scale bars: 10 mm (**A**); 10 µm (**B**); 50 µm (**C**–**E**); 2 µm (**F**).

**Figure 4:**
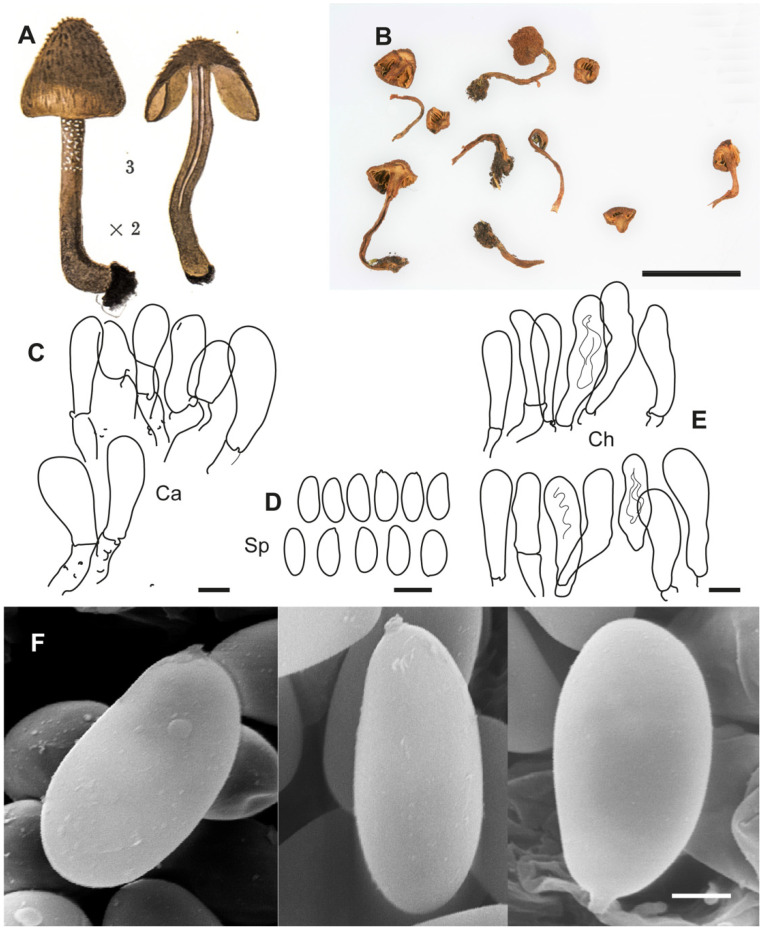
*Inocybe geraniodora* var. *gracilenta* Herb. J. Favre Z.A.82a (G 00551725). (**A**) Favre ([69]: pl. VI Figure 3). (**B**) Voucher material. (**C**) Caulocystidia. (**D**) Spores. (**E**) Cheilocystidia. (**F**) Spore SEMs. Scale bars: 10 mm (**B**); 10 µm (**C**–**E**); 2 µm (**F**).

Figure 3, Figure 4 and Figure 9D

= *Inocybe geraniodora* var. *gracilenta* J. Favre, Ergebnisse der Wissenschaftlichen Untersuchungen des Schweizerischen Nationalparks 5: 84, 1955. MycoBank No. 346937 (nom. inval., Art. 39.1).

**Diagnosis.** *Inosperma gracilentum* differs from other morphologically similar species such as *I. praetermissum* and *I. calamistratum* by the long ellipsoid spores, which are slightly concave and sometimes slightly phaseoliform in lateral view, and by the shorter cheilocystidia. It is phylogenetically distinct from all other species of the Calamistratum group.

**Holotype.** Sweden, Åsele lappmark, Vilhelmina, Klimpfjäll, Frimstjakke: 65°14′42.8″ N, 14°24′06.6″ E, alpine heath with *Dryas octopetala*, *Salix reticulata* and *Bistorta vivipara*, on calcareous ground, 1016 m alt., 22 August 2019, leg. E. Larsson, EL 85-19 (GB 0207620, isotype in AH 56238), GenBank accession: ITS-LSU (OR817726).

**Description.** *Pileus* 5–25 mm; when young, hemispherical, conical to obtuse conical with recurved margin; later, conico-convex to plano-convex; surface dry, margin fibrillose to finely scaly, centre of disc scaly to recurved scaly, ochraceous brown to reddish brown, velipellis ochraceous, sometimes not observed or only present in centre of pileus, fugacious. *Lamellae* rather sparse, broadly adnate to emarginate (L = 26–40), interspersed with lamellulae, initially pale beige, turning ochraceous brown with age, edge pale fimbriate. *Stipe* 15–30 × 1–3 mm, dry, equal to slightly bulbous, at the base bluish green, more ochraceous brown at apex, fibrillose, squamulose, flocculose at apex. *Context* ochraceous brown and bluish green at the base of stipe and in the middle of the pileus, more or less reddish on the upper part of the stipe when cut. *Odour* distinctly of pelargonium or fishy. *Basidiospores* variable, (10.2–)10.8–13.7(–14.4) × 6.0–7.4(–7.7) µm, Sp_avg_ = 12.1 × 6.5 µm, Q = (1.63–)1.70–2.04(–2.08), Q_avg_ = 1.8 (n = 67/1), smooth, ochraceous brown, long ellipsoid, some adaxially plane to slightly concave, typically depressed in the supra-apicular region (Figure 3B,F), with obtuse apex, apiculus small and not distinct. *Basidia* (31.2–)34.3–50.6(–52.5) × (9.0–)9.6–12.0(–12.1) µm, Ba_avg_ = 42.4 × 11.0 µm (n = 15/1), narrowly clavate, four-spored, hyaline. *Pleurocystidia* absent. *Cheilocystidia* (21.3–)22.7–41.7(–44.6) × (9–)9.6–14.1(–15.9) µm, Ch_avg_ = 30.4 × 11.8 µm, (n = 25/1), mostly pyriform to clavate, less often subcylindrical, hyaline or full or brownish-green pigment, thin-walled. *Caulocystidia* present near apex, like cheilocystidia but generally shorter, 20–42 × 9–16 µm (n = 35/1). *Clamp connections* present.

**Distribution.** Known only from the alpine areas of Europe in Sweden and Switzerland, where its presence is confirmed by molecular data. Its distribution range may be wider; however, the species seems to be rare, and few confirmed collections are known. There are no ITS sequences in GenBank nor in the UNITE database that match or are close to the samples studied.

**Ecology.** Found growing in the alpine zone on calcareous soils among *Salix reticulata*, *S. retusa*, *Dryas octopetala* and *Bistorta vivipara*.

**Etymology.** Refers to the Latin word *gracilentus*, which means slender, thin.

**Additional specimens examined.** Switzerland, Grisons, National Park, between Sur il Foss and Alp Minger: 46°42′31″ N, 10°15′31″ E, calcareous soil in a *Salix retusa* carpet, 2250 m alt., 17 August 1951, Herb. J. Favre Z.A.82a (G 00551725), GenBank accession: ITS1 (PP431544), ITS2 (PP431548).

**Notes**. Favre ([69]: 84) very briefly described a “varieté gracilenta” for *Inocybe geraniodora*, and the voucher collection (Figure 4) was examined by us (G!). This variety was not validly published by Favre because no Latin diagnosis was given (nom. inval., Art 39.1). Favre’s collection, based on the molecular data obtained, represents the same taxon as the Swedish holotype proposed here. It is a distinct species from *Inosperma geraniodorum*.

The holotype includes many specimens that have been examined in detail for their macro- and micromorphological characters, and ITS-LSU sequences were successfully obtained. *Inosperma gracilentum* is similar to *I. praetermissum* in morphological characters and habitat. It differs in the spores, which are broader with an ellipsoid and more regular outline, often adaxially slightly concave to hardly subphaseoliform, often depressed in the supra-apicular region (Figure 3B,F). The cheilocystidia are also much shorter in *I. gracilentum*, barely exceeding 35(–40) µm in length (Figure 3D). From the available data, it seems to be a rare species, and to us it is only known from two alpine localities in Europe. *Inosperma praetermissum* is more widespread in the Northern Hemisphere in the boreal zone and is also known from North America and Asia. *Inosperma gracilentum* can also resemble *I. subhirsutum* and the two can easily be confused, but they are genetically distinct (Figure 1). Both show a different appearance of the pileus and stipe surface, which is often more fibrillose and lanose (“mallocyboid”) in *I. subhirsutum* and more hirsute–strigose in *I. gracilentum*. Both can be separated in terms of micromorphology by the spore measurements, since *I. gracilentum* has narrower spores (Q_avg_ = 1.8) than *I. subhirsutum* (Q_avg_ = 1.6). *Inosperma subhirsutum* has more regular ellipsoid to ovo-ellipsoid spores, often flattened in profile (more reminiscent of the spores of *I. geraniodorum*), it has longer cheilocystidia and is collected more frequently and regularly in the alpine zone of the Alps and Fennoscandia.

***Inosperma neohirsutum* Esteve-Rav., Pancorbo & E. Larss., sp. nov.**

MycoBank No. 850656

**Figure 5:**
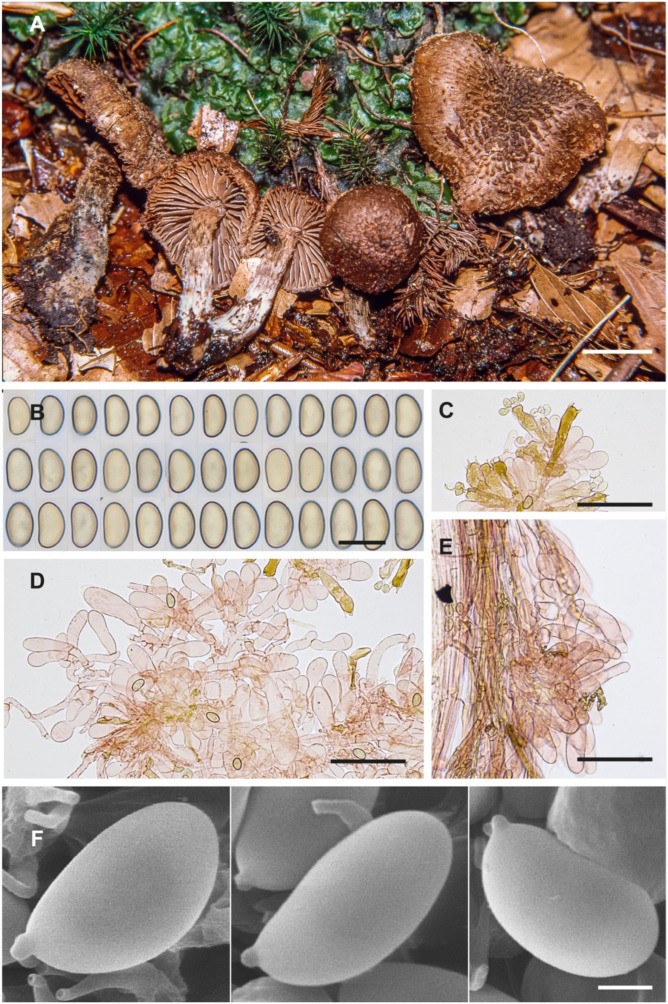
Inosperma neohirsutum (Holotype AH 26947 for (**A**–**E**), AH 48235 for (**F**)). (**A**) Basidiomata. (**B**) Basidiospores. (**C**) Basidia. (**D**) Cheilocystidia. (**E**) Caulocystidia. (**F**) Spore SEMs. Scale bars: 10 mm (**A**); 10 µm (**B**); 50 µm (**C**–**E**); 2 µm (**F**).

Figure 5 and Figure 9E–H

**Diagnosis**. *Inosperma neohirsutum* is similar to *I. calamistratum*, but differs from it due to its smaller size, less coriaceous flesh and a strongly hirsute scaly pileus on the central disc, which, as it develops, forms a characteristic squarrose patch of thick recurved, agglutinated and welded scales, which contrasts sharply with the fibrillose margin. The spores are also broader (Q_avg_ = 1.8). The closest ITS sequences in BLAST correspond to the American species *Inosperma apiosmotum* (Grund & D.E. Stuntz) Matheny & Esteve-Rav. with 94% similarity.

**Holotype.** Spain, Castilla y León, Segovia, Riofrío de Riaza, Puerto de La Quesera, Hayedo de La Pedrosa: 41°12′59.59″ N, 3°24′26.70″ W, in very humid soil among mosses in *Fagus sylvatica* forest, on acid granitic soil, 1700 m alt., 1 September 2001, leg. F. Esteve-Raventós & M. Villarreal, AH 26947 (isotype in GB: 0266843), GenBank accession: ITS (PP431510), LSU (PP431532).

**Description.** *Pileus* 15–25(–30) mm; when young, hemispherical convex to campanulate, sometimes obtusely conical, with deflexed, wavy margin; later, conico-convex to plano-convex, sometimes with a slightly depressed centre; dry, not hygrophanous, margin crenate; surface fibrillose–lacerate to finely scaly towards the edge, strongly scaly and hirsute–squarrose on the disc, formed by clusters of recurved concolourous scales, these often aggregated and coarse in the centre, sometimes appearing as pyramidal aggregates with age. Colour uniformly brown to chocolate brown on a lighter background as the surface breaks into scales; velipellis not observed. *Lamellae* moderately dense (L = 30–40), narrowly adnate to emarginate, subventricose, interspersed with numerous lamellulae l = 1–2(–3), initially pale beige, turning ochraceous brown with age, becoming concolourous to pileus, edge pale, fimbriate. *Stipe* 15–35(–40) × (2.5–)3–5 mm, dry, cylindrical, tapering upwards to subclavate; with age, brown to concolourous to pileus, usually bluish green at base but sometimes absent or very pale, apex ochraceous to brown; surface regularly squamulose to squarrose, covered with concolourous recurved scales, apex only flocculose. *Context* pale ochraceous to buff, hardly greenish to glaucous at the base of stipe, slightly reddening on the upper part of the stipe when cut. *Odour* sometimes faint, aromatic, fruity–acidic, sometimes with a scent of pelargonium or fish. *Basidiospores* (8.2–)9.0–10.9(–12.0) × (4.5–)5–6.1(–6.3) µm, Sp_avg_ = 9.9 × 5.5 µm, Q = (1.54–)1.61–2.01(–2.23), Q_avg_ = 1.8 (n = 307/3), smooth, ellipsoid, phaseoliform in profile, ochraceous brown. *Basidia* 36.1–50.0(–56.4) × 9.1–13.7(–14.1), Ba_avg_ = 42.3 × 11.0 µm, clavate, mainly four-spored, hyaline, often with intracellular brown pigment. *Pleurocystidia* absent. *Cheilocystidia* rather short, (16.5–)23.3–53.0(–68.2) × (8.3–)8.9–15.9(–17.5) µm, Ch_avg_ = 35.2 × 12.0 µm (n = 133/3), mostly broadly clavate to spheropedunculate, less often subclavate to subcylindrical, sometimes subcapitate, usually one- to two-septate at the base and then resembling the *Opuntia*-like arrangement of cheilocystidia in certain *Mallocybe* spp. *Caulocystidia* present near stipe apex, grouped in clusters, similar to cheilocystidia, (19.3–)19.6–42.0–65.2(–67.1) × (6.6–)7.6–11.2–15.2(–17.1) µm, Ca_avg_ = 42.0 × 11.2 µm, (n = 28/2). *Clamp connections* present.

**Distribution.** The distribution of *I. neohirsutum* on the European continent is still unclear. It is very likely that some European records of *I. calamistratum* represent *I. neohirsutum*. To date, its presence has been confirmed in France, Norway, Sweden and Spain. However, according to the available distribution data and habitat preferences, it is likely to be widespread throughout the continent, thriving in humid forests in mainly temperate to mild climates of continental, Atlantic and hemiboreal type. It is unlikely that it will be found in colder climates of the northern boreal and alpine zones. There is only one sequence available matching *I. neohirsutum*, in the UNITE database, from Arendal, Norway, collected under *Quercus* and *Corylus* (UDB07673483/NOBAS 1849-16, as *Inocybe calamistrata*).

**Ecology.** It grows in both moist broadleaved forests of *Fagaceae* (*Fagus* and *Quercus*) and *Betulaceae* (*Corylus*) and mixed conifer-dominated forests, either in acidic or calcareous soils.

**Etymology.** From Greek *néos*, meaning new, and Latin *hirsus*, meaning hirsute, hairy, shaggy. Refers to a new or different interpretation of *Inocybe hirsuta*.

**Additional specimens examined.** France, Nouvelle-Aquitaine, Pyrénées-Atlantiques, Osse-en-Aspe, Forêt d’Issaux: 42°59′44″ N, 0°41′51″ W, in very humid soil among mosses in *Fagus sylvatica* forest in calcareous soil, 1044 m alt., 13 October 2018, leg. F. Pancorbo, AH 48235 (dupl. FP 18101301), GenBank accession: ITS (PP431512), LSU (PP431533). Spain, Castilla y León, Segovia, Riofrío de Riaza, Puerto de La Quesera, Hayedo de La Pedrosa: 41°12′59.59″ N, 3°24′26.70″ W, in very humid soil among mosses in *Fagus sylvatica* forest, on acid, granitic soil, 1700 m alt., 9 September 1986, leg. F. Esteve-Raventós, G. Moreno & C. Illana, AH 09624. Ibidem: 1 October 1989, leg. F. Esteve-Raventós & A. Altés, AH 18865. Ibidem: 16 October 1996, leg. F. Esteve-Raventós & M. Villarreal, AH 21333. Ibidem: 22 September 1993, leg. F. Esteve-Raventós & M. Heykoop, AH 22146. Ibidem: 14 September 1990, leg. F. Esteve-Raventós, G. Moreno & M. Heykoop, AH 24593, GenBank accession: ITS (PP431511). Ibidem: 27 August 1995, leg. P. Juste, AH 24959 (dupl. in Herb. Asociación Micológica de Tudela de Duero n° 1180). Sweden, Västergötland, Östad, Risveden, Långevattnet: in mixed coniferous forest close to *Pinus sylvestris* and *Betula pendula* on acidic soil, 20 September 2015, leg. E. Larsson, EL 163-15 (GB 0207661), GenBank accession: ITS-LSU (OR831119), RPB2 (PP092169). Västergötland, Ödenäs, close to the church: in mixed coniferous forest on acidic soil, 17 October 2003, leg. E. Larsson, EL 77-03 (GB 0150442), GenBank accession: ITS-LSU (AM882945)—as *Inocybe* cf. *calamistrata*.

**Notes**. *Agaricus hirsutus* was described by Lasch [70] with a succinct description that could apply to both *I. calamistratum* and *I. neohirsutum*. The habitat is noted as “in fagetis humidis”, a habitat that may support both species. Kuyper [4] considered *Inocybe hirsuta* to be a synonym of *I. calamistrata*, a treatment with which we agree, since it is impossible to separate the two species with the available data from the protologues. It is very likely that among the numerous records of *I. calamistratum* in Europe, some of them could correspond to *I. neohirsutum*. In our study, molecular analysis has confirmed the existence of two distinct species with rather similar morphological characters.

One of the macroscopic differences between the two species mostly lies in the size of the basidiomata. *Inosperma calamistratum* often produces larger basidiomes, with a normally long, fibrous and elastic stipe, whereas *I. neohirsutum* is smaller, with a more fragile stipe (Figure 9E–H). It is not improbable to assume that Lange’s [71] interpretation of *Inocybe calamistrata* f. *gracilis* J.E. Lange (nom. inval., art. 36.1) may correspond, at least in part, to *I. neohirsutum.* However, a studied collection (AH 58564) under *Abies* and *Fagus* from Aragón (Spain) showed a small size reminiscent of f. *gracilis* and is in molecular agreement with *I. calamistratum*. In these cases, to separate *I. calamistratum* and *I. neohirsutum*, it is necessary to analyse other morphological characters, such as the appearance of the pileus surface, the cheilocystidia, and the spore shape and size.

*Inosperma neohirsutum* is probably not an uncommon species from the humid, temperate forests of Europe, in both deciduous and coniferous forests. So far, it has been overlooked or probably misinterpreted as a smaller form of *I. calamistratum*. It has a very peculiar macroscopic character, namely, the squarrose aspect (reminiscent of *Inocybe hystrix*, for example) on the central disc of the pileus, which contrasts sharply with the more fibrillose to subsquamose margin, especially in adult specimens when the pileus is fully extended (Figure 5A and Figure 9E–H). In *I. calamistratum*, the scales are very abundant, dense, sharp, thinner and mostly distributed over the whole surface of the pileus. The shape of the spores also differs between the two species, and although phaseoliform in both, this characteristic is somewhat less pronounced in *I. neohirsutum* (Figure 5B,F), where the Q_avg_ is smaller (1.8 vs. 2.0). The trend in the morphology of the cheilocystidia of the two species also appears to be different, being narrower and subcylindrical in *I. calamistratum*. Because of its habitat, *I. neohirsutum* cannot be confused with other similar northern boreal and alpine species, such as *I. gracilentum*, *I. subhirsutum* and *I. praetermissum*.

**Figure 6:**
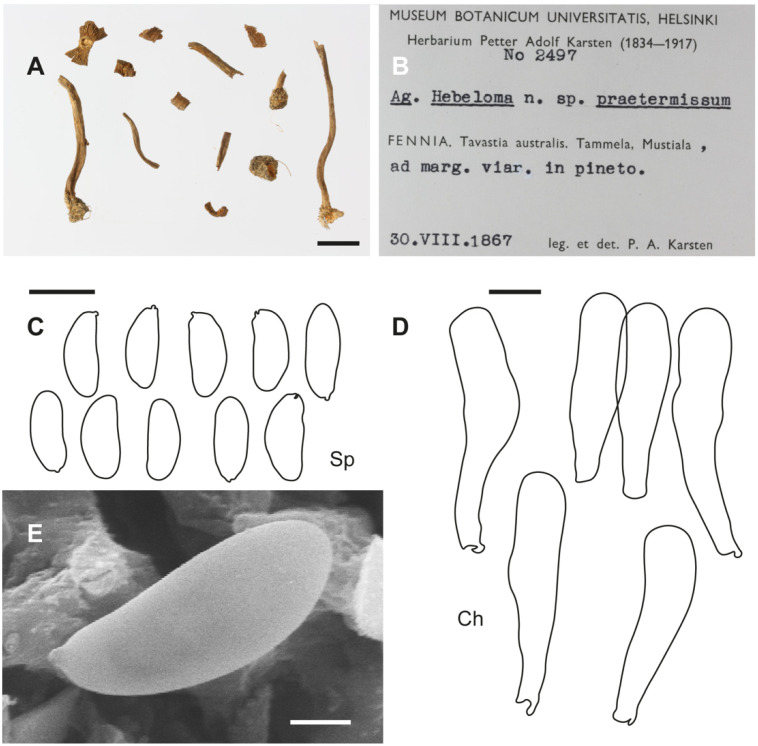
*Inosperma praetermissum* Holotype Herb. P.A. Karsten 2497 (H). (**A**,**B**) Voucher material and label. (**C**) Basidiospores. (**D**) Cheilocystidia. (**E**) Spore SEM. Scale bars: 10 mm (**A**); 10 µm (**C**,**D**); 2 µm (**E**).

In our phylogenetic analysis, *I. neohirsutum* appears to be closely related to *I. subhirsutum*, which has a lower Q_avg_ of the spores, showing an ovoid to broadly ellipsoid outline, and to the North American species *Inosperma apiosmotum* (Grund & D.E. Stuntz) Matheny & Esteve-Rav., which is also small to medium in size and emits a typical odour of ripe pears. Grund and Stuntz [72] already noted in their observations its resemblance to *Inocybe calamistrata* and *I. hirsuta*. *Inosperma apiosmotum* and *I. neohirsutum* show similarities in the arrangement of scales on the pileus ([72], Figure 2 and Figure 6) and in microscopic characters, such as the shape and size of spores and cheilocystidia.

***Inosperma praetermissum* (P. Karst.) Esteve-Rav., E. Larss. & Pancorbo, comb. nov.**

MycoBank Transfer No. 850526

**Figure 7:**
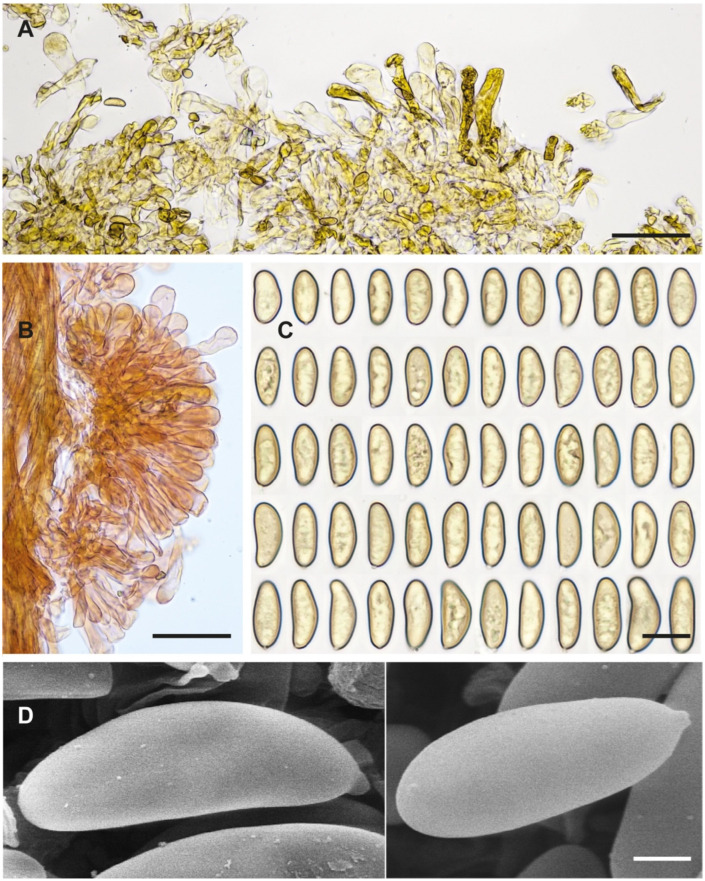
*Inocybe geraniodora* var. *gracilenta* f. *salicis-herbaceae* Holotype JB 1620/95. (**A**) Cheilocystidia. (**B**) Caulocistydia. (**C**) Basidiospores. (**D**) Spore SEMs. Scale bars: 50 µm (**A**,**B**); 10 µm (**C**); 2 µm (**D**).

Figure 6, Figure 7 and Figure 10A–C

≡ *Inocybe praetermissa* P. Karst., Meddelanden af Societas pro Fauna et Flora Fennica 11: 3, 1885. MycoBank No. 239808

= *Inocybe geraniodora* var. *gracilenta* f. *salicis-herbaceae* Bon & Ballarà, Revista Catalana de Micologia 19: 145, 1996. MycoBank No. 446932

**Holotype.** Finland, Tavastia Australis, Tammela, Mustiala: under *Pinus* on a roadside, 30 Aug, 1867, Herb. P.A. Karsten 2497 (H), GenBank accession: ITS2 (PP431551).

**Description.** Karsten [73], Breitenbach and Kränzlin ([57], photo as *Inocybe calamistrata*, no. 8), Jamoni ([74], as *Inocybe calamistrata*), Bon and Ballarà ([75], as *Inocybe geraniodora* var. *gracilenta* f. *salicis-herbaceae*), Armada et al. ([76], as *Inosperma* cf. *calamistratum*).

**Additional microscopic examination of the holotype of *I. praetermissum.*** The details given are mainly based on T.W. Kuyper’s revision and annotations in 1984 and our supplementary information. Caulocystidia were not examined due to the scarcity of material. *Basidiospores* (10.6–)11.2–13.1(–14.2) × 5.1–5.7(–6.0) µm, Sp_avg_ = 12.2 × 5.3 µm, Q = (1.9–)2.1–2.6), Q_avg_ = 2.2 (n = 30), [Kuyper: (10.0–)10.5–12.0(–13.0) × 5.0–5.5(–6.0) µm, Q = (1.9–)2.0–2.4(–2.6)] smooth, thin-walled, ellipsoid, subphaseoliform in profile, yellowish greenish. *Basidia* clavate, mainly four-spored, hyaline. *Pleurocystidia* absent. *Cheilocystidia* (30–)35–55(–60) × 8–11 µm, cylindrical, subclavate or clavate, sometimes flexuose, with rounded apex, hyaline, thin-walled. *Clamp connections* present.

**Additional microscopic examination of the holotype of *I. geraniodora* var. *gracilenta* f. *salicis-herbaceae*.** *Basidiospores* (10.6–)11.6–14.5(–15.8) × (4.8–)5.0–6.2(–7.0) µm, Sp_avg_ = 13.2 × 5.5 µm, Q = (1.92–)2.05–2.68(–2.94), Q_avg_ = 2.3 (n = 92), smooth, thin-walled, elongated ellipsoid to bacilliform, subphaseoliform in profile. *Basidia* clavate, predominantly four-spored. *Pleurocystidia* absent. *Cheilocystidia* (44.3–)45.8–63.5(–63.9) × (9.5–)10.4–16.6(–16.7) µm, Ch_avg_ = 54.8 × 13.0 µm (n = 20), cylindrical, subclavate, with rounded apex, with brown-greenish pigment, thin-walled. *Caulocystidia* present near apex, (51.2–)51.9–68.2 × (10.8–)11.5–16.0(–16.8) µm, Ca_avg_ = 60.2 × 14.3 µm (n = 13) clustered, cheilocystidia-like. *Clamp connections* present.

**Distribution.** The sequenced collections studied by us and those deposited in GenBank suggest a wide distribution of *I. praetermissum* on the European continent, always in cold bioclimates, whether boreal–alpine, subalpine, altimontane or hemiboreal. Its distribution ranges from Greenland and the Nordic countries to the Pyrenees as the southernmost limit. In addition to the sequences from Finland (where it was originally found), the Alps and the Pyrenees, there are other sequences deposited in GenBank from the Swiss Alps (MK838291 and MT095693). It also occurs in cold mountainous areas of the northeastern Czech Republic (OM793002) in the Giant Mountains, under *Picea* (M. Vasutova, pers. comm.). In North America, *I. praetermissum* also seems to occur in similar boreal-alpine ecosystems and montane coniferous forests (JQ801386 and OQ701112). Several sequences (clones) from alpine meadows in China with the presence of *Polygonum* and *Kobresia* (FJ827203, FJ378766 and OL850876) may also correspond to *I. praetermissum*.

**Ecology.** On both calcareous and acidic soils, under conifers (*Pinus*, *Picea*, *Abies* and *Tsuga)* and *Betula pubescens* in montane–subalpine and hemiboreal areas, and in the alpine zone with shrubs or herbaceous plants (*Salix herbacea*, *S. retusa*, *S. lapponum*, *Dryas* spp., *Betula nana*, *Bistorta vivipara* and *Kobresia*).

**Etymology.** From the Latin *praetermissus*, meaning overlooked, neglected.

**Additional specimens examined.** Andorra, Arcalís: in alpine area dominated by *Salix herbacea*, 2350 m alt., 26 August 1995, leg. J. Ballarà, holotype of *I. geraniodora* var. *gracilenta* f. *salicis-herbaceae*, JB 1620/95 (isotype in AH 46901), GenBank accession: ITS1 (PP431546), ITS2 (PP431550). Italy, Trentino-Alto Adige, Passo Venegiotta, at the foot of Cima Mulaz: 46°19′35″ N, 11°49′38″ E, in calcareous *Salix retusa* and *S. reticulata* scrub, 2314 m alt., 30 August 2005, leg. E. Bizio, EB 2005083005 (dupl. AH 56197). Trentino-Alto Adige, Parco di Paneveggio, Pale di San Martino, Malga Juribrutto: 46°19′36″ N, 11°46′51″ E, in *Picea abies* forest, on quartz–porphyry calcareous soil, 1800 m alt., 17 August 2019, leg. E. Bizio, EB 2019081705 (dupl. AH 46960), GenBank accession: ITS (PP431527). Trentino-Alto Adige, Parco di Paneveggio, Baita Segantini, Passo Rolle: 46°17′53″ N, 11°48′18″ E, in calcareous alpine scrubland, 2200 m alt., 30 August 2019, leg. E. Bizio, EB 2019083003 (dupl. AH 46963), GenBank accession: ITS (PP431528). Trentino-Alto Adige, Pale di Gerda: 43°32′17″ N, 11°58′48″ E, in calcareous alpine scrubland, 2250 m alt., 24 August 2019, leg. R.J. Ferrari, AH 46977 (dupl. FRJ 036-2019). Spain, Cataluña (Catalonia), Girona, Vall de Núria, ras de l’Ortigar: 42°23′46″ N, 2°08′40″ E, in calcareous alpine scrubland under *Dryas octopetala* and *Salix retusa*, 2220 m alt., 10 August 1999, leg. J. Vila, JVG 990810-3 (dupl. AH 26722). Sweden, Bohuslän, Grinneröd, Norra fjället: in mixed forest close to *Picea abies* and *Corylus avellana* on acid soil, 6 July 2014, leg. E. Larsson, EL 4-14 (GB 0243035), GenBank accession: ITS-LSU (OR831122). Bohuslän, Uddevalla, Kuröds skalgrusbankar: under *Betula pendula* and *Corylus avellana*, 2 October 2004, leg. E. Larsson, EL 130-04 (GB 0240811), GenBank accession: ITS-LSU (AM882944)—as *Inocybe calamistrata*. Bohuslän, Resteröd, Ulvesund, Grinddalen: in mixed coniferous forest close to *Picea abies* and *Corylus avellana* on acid soil, 2 October 2004, leg. E. Larsson, EL 139-04 (GB 0240820). Torne lappmark, Jukkasjärvi, Abisko, along Rakkasjokka: alpine heath with *Salix herbacea* and *Bistorta vivipara*, 24 August 2013, leg. J. Vauras, EL 206-13 (GB 0243036), GenBank accession: ITS-LSU (OR831121), RPB1 (PP092180), RPB2 (PP092171). Åsele lappmark, Vilhelmina, Fiehteres: alpine heath, snowbed area with *Salix herbacea*, 1070 m alt., 21 August 2019, leg. E. Larsson, EL 70-19 (GB 0243039), GenBank accession: ITS-LSU (OR831123). Pite lappmark, Arjeplog, northeast side of Ákharis: alpine heath with *Salix* spp. and *Betula nana*, 14 August 2018, leg. J. Vauras, EL 161-18 (GB 0243037), GenBank accession: ITS-LSU (OR831120), RPB2 (PP092170). Jämtland, Frostviken, Raavre: alpine heath, moist with *Salix herbacea* and *Bistorta vivipara*, 800 m alt., 23 August 2019, leg. J. Vauras, EL 130-19 (GB 0243038), GenBank accession: ITS-LSU (OR831124).

**Notes**. *Inosperma praetermissum* is morphologically similar to *I. calamistratum*, *I. gracilentum* and *I. subhirutum*. After the revision of Karsten’s type, Kuyper [4] considered *I. praetermissum* to be a synonym of *I. calamistratum*. However, the molecular study has allowed for a distinction between the two species. *Inosperma praetermissum* is relatively common in the hemiboreal, subalpine and alpine areas of the Northern Hemisphere and has probably been interpreted as a form or ecological variant of *I. calamistratum*. The latter prefers more temperate and very humid ecosystems, either continental or Atlantic, either mesophilic or montane, but the two co-occur in the hemiboreal zone in Fennoscandia. Apart from being genetically different, *I. praetermissum* can be separated, as it is smaller and more fragile in appearance than *I. calamistratum*. *Inosperma praetermissum* shows a clear tendency to have a pileus not as squarrose as that of *I. calamistratum*, with paler, woolly–fibrillose and looser scales that are not as prominent or as sharply defined, especially in the centre of the pileus (Figure 10A–C). Also often observed is the presence of a persistent ochraceous veil, resembling a fibrillose covering. In addition, the cheilocystidia are slightly longer than those of *I. calamistratum*, with a more elongated claviform outline, usually reaching 60 µm in length (Figure 6D). Care should be taken when observing the blue-greenish colour of the stipe and when perceiving the odour, as both can sometimes go unnoticed or blurred, especially if the specimens are old or soaked after heavy rain. Micromorphologically, the spores (Figure 6C,E) are somewhat similar in appearance to those of *I. calamistratum*, being subphaseoliform to phaseoliform in profile, although they are longer on average and often appear narrowly phaseoliform (12.2 × 5.3 µm vs. 10.4 × 5.1 µm; Q_avg_ = 2.2 vs. 2.0). The odour of *I. praetermissum* was described by Karsten as unpleasant (“inamoenus”) and strong (“gravis”) and has been noticed in some specimens as smelling like fish brine.

Bon [5] mentioned the possible presence (most probably in the Alps) of *Inocybe praetermissa* (s.str. P. Karst.) at subalpine levels under conifers and *Vaccinium* bushes, to which he attributes a pileus surface not as hirsute as in *I. calamistratum*, an odour that sometimes eventually develops into that of *I. cervicolor* (earthy–mouldy), and elongated, narrow spores 12–14(–15) × 5–6(–7) µm. This interpretation agrees with the data obtained in the collections studied.

*Inosperma gracilentum*, which also occurs in alpine and boreal areas, can be confused with *I. praetermissum*, but the former is distinguished by its molecular characteristics, shorter cheilocystidia barely exceeding 40 µm in length, and the more ellipsoidal and somewhat broader spores (Sp_avg_ 12.2 × 5.3 µm, Q_avg_ = 2.2 vs. Sp_avg_ 12.1 × 6.5 µm, Q_avg_ = 1.8).

*Inosperma subhirsutum* collections growing in hemiboreal mixed *Alnus* forests and in subalpine *Betula* forests often have larger basidiomata than the average for the alpine zone, and they can then be confused in their macromorphology with *I. pratermissum*, but the two are clearly separated by spore morphology.

DNA extraction was successful in the holotype of *Inocybe geraniodora* var. *gracilentum* f. *salicis*-*herbaceae*, despite its poor condition. Based on the characters described in the protologue and on the study of the holotype (Figure 7), we consider it a synonym of *I. praetermissum*. The holotype of *I. praetermissum* shows slightly smaller and narrower spores (L/l_avg_ 12.2 × 5.3 vs. 13.4 × 5.7; Q_avg_ 2.2 vs. 2.3), although the other characters overlap or coincide in both. As in the case of *I. geraniodora* var. *gracilenta* f. *salicis-herbaceae*, the shapes of the spores of *I. praetermissum* are narrowly subphaseoliform in profile, and the cheilocystidia share similar dimensions and morphology. Finally, the phylogenetic study (ITS) indicates that both taxa are cospecific. The odour was also described as unpleasant and fishy.

***Inosperma subhirsutum* (Kühner) Matheny & Esteve-Rav., Mycologia 112(1): 105, 2019.**

MycoBank No. 830402

**Figure 8:**
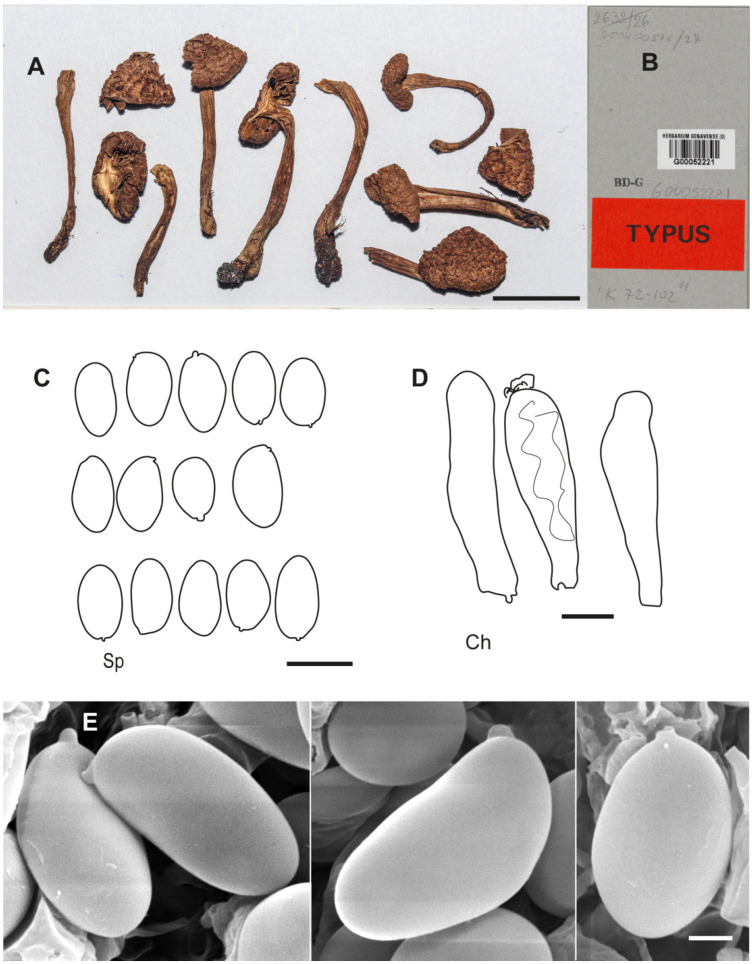
*Inosperma subhirsutum* Holotype Herb. R. Kühner 72-102 (G00052221). (**A**,**B**) Voucher material and label. (**C**) Basidiospores. (**D**) Cheilocystidia. (**E**) Spore SEMs. Scale bars: 10 mm (**A**); 10 µm (**C**,**D**); 2 µm (**E**). Photographs (**A**,**B**) by J.C. Zamora.

Figure 8 and Figure 10D–F

≡ *Inocybe subhirsuta* Kühner, Documents Mycologiques 19(74): 25, 1988. MycoBank No. 135080

**Holotype.** France, Savoie, Parc National de la Vanoise, Haute Maurienne, Plan des Évettes: 45°21′48.93″ N, 7°6′43.04″ E, under *Salix reticulata* in calcareous soil, 2500 m alt., 12 August 1972, Herb. R. Kühner 72-102 (G 00052221), GenBank accession: ITS (PP065739).

**Description.** Kühner [77], Bon [65].

**Additional microscopic examination of the holotype of *I. subhirsutum.*** *Basidiospores* 10.7–13.0(–13.7) × 6.2–7.8 µm, Sp_avg_ = 11.8 × 7.0 µm, Q = (1.44–)1.46–1.85, Q_avg_ = 1.6 (n = 21), smooth, thin-walled, variable in shape, ovo-ellipsoid to ovo-subamygdaliform, broadly ellipsoid to ellipsoid, often somewhat flattened in profile view, rarely subphaseoliform. *Basidia* clavate, mainly four-spored, hyaline, often with greenish-brown pigment. *Hymenial trama* greenish brown. *Pleurocystidia* absent. *Gill edge* heterogeneous. *Cheilocystidia* (35–)37.8–45.3 × 9.1–13.0 µm, Ch_avg_ = 41.1 × 11.1 µm (n = 3), cylindrical, narrowly clavate to clavate, with rounded apex, hyaline, often with olive greenish content, thin-walled. *Caulocystidia* present near apex, grouped in clusters, cheilocystidia-like. *Clamp connections* present.

**Distribution.** *Inosperma subhirsutum* was first found in France in the alpine zone. It is more than likely that the species thrives at high altitudes in the Alps, as we have found by studying several samples from Italy. It is logical to assume its presence in other European countries with alpine ranges, such as Switzerland, Austria, etc., of which we have no records to date. Its presence in the Pyrenees is not known yet. According to the data collected by Jacobsson and Larsson [78], the species seems to be frequent and widely distributed in the boreal and alpine zones of Fennoscandia and Iceland. This is confirmed by several collections with sequences deposited both in GenBank (AM882946 and AM882947) and the UNITE database (UDB001195 and UDB07673483).

**Ecology.** In Europe, it grows in alpine, subalpine and boreal areas, mainly on calcareous soils, but also sometimes on neutral or slightly acidic soils. It occurs mainly in communities of various shrubs and small *Salicaceae*, sometimes mixed with *Betula nana or Bistorta vivipara*. In calcareous soils, it is most often associated with *Salix reticulata*, *S. retusa*, *S. glauca* and *Dryas octopetala.* It can also be found with *Salix herbacea* on soils with a more acidic component or of a sandy nature due to soil washing. It is known to occur in the Alps in subalpine areas (e.g., in Lago Dobbiaco, Italy, EB 20140807), close to *Salix* near watercourses in coniferous forests (*Picea*). In boreal areas, it can also be found in coniferous vegetation, associated with *Salix*, or in mixed forests with *Populus tremula, Alnus incana* and *Betula pubescens* (GB 0243043). The ectomycorrhizal relationship of *I. subhirsutum* with *Salicaceae* especially seems obvious, as well as its localisation in hemiboreal, subalpine and alpine zones.

**Etymology.** From the Latin *sub*-, meaning under, beneath, behind and near, and *hirsus* (variant of *hirtus*), meaning shaggy, rough, hairy, referring to the appearance of the pileus.

**Additional specimens examined.** Finland, Ostrobottnia ultima, Rovaniemi, Kylmäoja: moist area with *Populus tremula*, *Betula pubescens* and *Alnus incana*, 200 m alt., 3 September 2013, leg. E. Larsson, EL 352-13 (GB 0243043), GenBank accession: ITS-LSU (OR817728). Italy, Piemonte, Vercelli, Alagna Valsesia, Monte Rosa, Conca delle Pisse: 45°52′53.8″ N, 7°53′2″ E, in acidic soil under *Salix herbacea* shrub, 2515 m alt., 26 August 1992, leg. E. Bizio, EB 1992082601 (dupl. AH 56195), GenBank accession: ITS (PP431507), LSU (PP431530). Trentino-Alto Adige, Bolzano, Dobbiaco, Lago di Dobbiaco: 46°41′46″ N, 12°13′12″ E, in calcareous, sandy soil, among mosses on the edge of a torrent near *Salix* sp. and *Picea abies*, 1260 m alt., 7 August 2014, leg. E. Bizio, EB 2014080711 (dupl. AH 56193), GenBank accession: ITS (PP431509), LSU (PP431531). Norway, Sør-Trøndelag, Oppdal, Dovre, Kongsvoll: moist area with *Betula nana* and *Salix reticulata* on calcareous soil, 19 August 2012, leg. E. Larsson, EL 76-12 (GB 0243034), GenBank accession: ITS-LSU (OR817729), RPB2 (PP092163). Hordaland, Ulvik, Finse, Blåisen: snowbed area with and *Salix herbacea*, 1375 m alt., 11 August 2005, leg. E. Larsson, EL 26-05 (GB 0248023), GenBank accession: ITS2-LSU (AM882946), RPB1 (PP092179), RPB2 (PP092165)—as *Inocybe calamistrata*. Hordaland, Ulvik, Finse, Sandalsnut: alpine meadow on calcareous soil, with *Dryas octopetala* and *Salix reticulata*, 12 August 2005, leg. E. Larsson, EL 43-05 (GB 0248040), GenBank accession: ITS-LSU (AM882947)—as *Inocybe calamistrata*. Sweden, Jämtland, Åre, Mt Åreskutan BaseCamp: 63°25′42.78″ N, 13°4′45.04″ E, in *Salix herbacea* shrubland, 1250 m alt., 26 July 2018, leg. J.C. Zamora, AH 46825, GenBank accession: ITS (PP431508). Medelpad, Alnön, Storsjönäset: moist forest with *Betula pubescens* and *Salix* spp., 75 m alt., 12 September 2014, leg. E. Larsson, EL 142-14 (GB 0243040), GenBank accession: ITS-LSU (OR817727), RPB1 (PP092177), RPB2 (PP092162). Pite lappmark, Arjeplog, Årjep Rivatjåkkå, NE of Skärrim: moist alpine meadow on calcareous ground, under *Salix reticulata*, 15 August 2018, leg. E. Larsson, EL 198-18 (GB 0243041), GenBank accession: ITS-LSU (OR817730), RPB2 (PP092164). Åsele lappmark, Vilhelmina, Lasterfjället, Tjårronjunjes NV side: alpine heath on calcareous soil, with *Salix reticulata*, 19 August 2019, leg. E. Larsson, J.B. Jordal & J. Vauras, EL 11-19 (GB 0454414), GenBank accession: ITS-LSU (PP512979).

**Notes**. *Inosperma subhirsutum* is another hemiboreal to alpine species in the Calamistratum group. From its morphological and organoleptic characteristics, it seems to show some similarities with the species of the Geraniodorum group in terms of the appearance and shape of the pileus, its surfaces, and its odour reminiscent of pelargonium [77]. Its spores show a certain variability, even in the same collection, and are often flattened and sometimes slightly concave to subphaseoliform in profile view (Figure 8C,E). In frontal view, the spores are ovo-ellipsoid to ellipsoid and broader. According to our observations, the greenish-blue colour at the base of the stipe noted by Kühner in his original diagnosis may be variable, and some collections show a greenish grey to dark dirty-grey at the base, but this colour may be completely absent in some collections (EB 2014080711). Also noteworthy is the pelargonium-like odour in young specimens, which may become fishy later (according to Kühner, the odour is “reminiscent of herring”).

Characteristic of *I. subhirsutum* are the pileus and stipe coverings (Figure 10D,E), which are usually woolly–fibrillose (“mallocyboid”). However, some collections show a more hirsute pileus surface (e.g., EL 11-19, Figure 10F). The stipe is similar in appearance to the pileus, also woolly–fibrillose, and never hirsute or squarrose. We have observed a variable tendency to reddening of the flesh and, in some collections of young specimens, the presence of a yellowish tinge in the lamellae.

Kühner [77] provisionally named this species *Inocybe calamistrata* var. *latispora*, because the spores have a distinct ovoid–ellipsoid outline in frontal view, in contrast to *I. calamistratum* and other similar species, where narrower spores with subphaseoliform to phaseoliform outlines predominate. Only *I. gracilentum* can show spores of similar width to *I. subhirsutum*, but the Q_avg_ is higher (1.8–2.0), and therefore their appearance is more elongated. Kühner’s paper was published after the validation of the holotype, due to a delay in publication.

The numerous collections studied, especially those from Fennoscandia, where it seems to be common, indicate that the spore morphology of *I. subhirsutum* may show some variation in width and appearance. The holotype collection shows a clear dominance of broadly ellipsoidal to ovo-ellipsoidal spores in frontal view, but this feature is variable even within the same collection. Kühner [77] reported spore dimensions of “9–10.6–13.5–14.7 × 6.2–6.7–8.2–9 µm, Q = 1.4–1.7”, which is in reasonable agreement with our examination of the holotype. The holotype was originally collected in an alpine zone under *Salix reticulata* on calcareous soil (“in calcareo solo”, according to Kühner), but we have also studied genetically matching samples from more acidic soils (probably washed) with *Salix herbacea*. These latter may also show some variability in spore size and shape, also including spores that are slightly narrower and more ellipsoidal than those reported for the holotype. Unfortunately, we were only able to observe a few cheilocystidia in Kühner’s collection, as the lamellar edge was quite collapsed, and the dimensions given here are only approximate. Very interesting are the data reported by Kuyper [79] from a collection in Jämtland (Sweden) under *Salix retusa* in the boreal zone; although he called it *I. calamistrata*, considering it and *I. subhirsuta* as synonyms, it seems to correspond to the latter because of its wider spores of 6–6.5 µm and its habitat. Phylogenetic studies support the separation of the two species at the specific level.

As can be observed from the phylogenetic tree (Figure 1), there is a rather large amount of genetic variation within *I. subhirsutum*, and we can genetically regard it as a species complex. There is a tendency to separate the strict Arctic–alpine specimens associated with dwarf *Salix* and *Dryas* and those from the boreal areas associated with *Populus tremula*, *Alnus*, *Betula pubescens* and mixed *Pinus*–*Betula* habitats. However, more data are needed to confirm this hypothesis to determine whether genetic data can be correlated with the observed morphological variation in, e.g., spore morphology and habitats.

**Figure 9:**
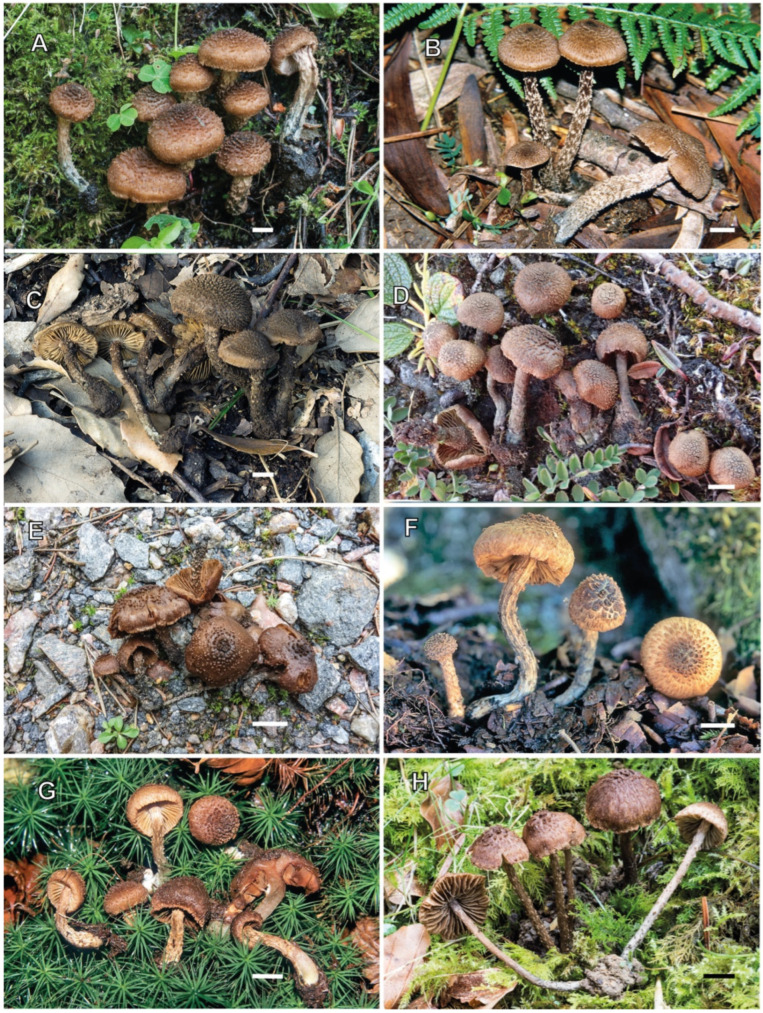
Basidiomes of the species of the Calamistratum group. (**A**) *Inosperma calamistratum* AH 44420 (ESP). (**B**) *I. calamistratum* AH 46636 (POR). (**C**) *I. calamistratum* AH 56397 (ESP). (**D**) *I. gracilentum* Holotype EL 85-19 (SWE). (**E**) *I. neohirsutum* EL 163-15 (SWE). (**F**) *I. neohirsutum* AH 24959 (ESP). (**G**) *I. neohirsutum* AH 21333 (ESP). (**H**) *I. neohirsutum* AH 48235 (FRA). Photograph (**C**) by E. Rubio, (**F**) by P. Juste. Scale bars: 10 mm (**A**–**H**).

**Figure 10:**
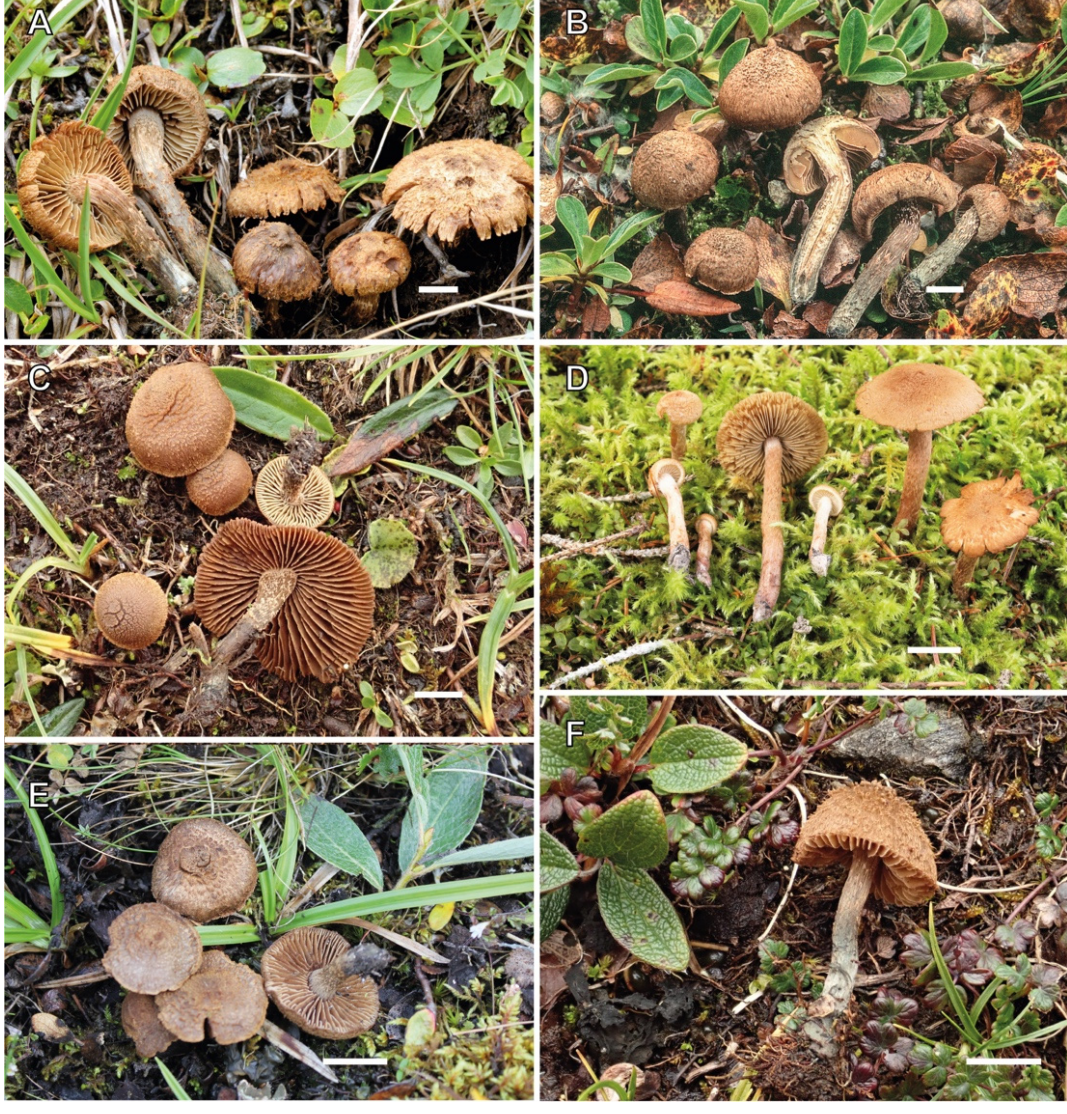
Basidiomes of Calamistratum group. (**A**) *Inosperma praetermissum* AH 46960 (ITA). (**B**) *I. praetermissum* EB 2005083005 (ITA). (**C**) *I. praetermissum* EL 130-19 (SWE). (**D**) *I. subhirsutum* EB 2014080711 (ITA). (**E**) *I. subhirsutum* EL 76-12 (SWE). (**F**) *I. subhirsutum* EL 11-19 (SWE). Scale bars: 10 mm (**A**–**F**).

***Inosperma geraniodorum* (J. Favre) Matheny & Esteve-Rav., Mycologia 112(1): 102, 2019.**

MycoBank No. 830366

**Figure 11:**
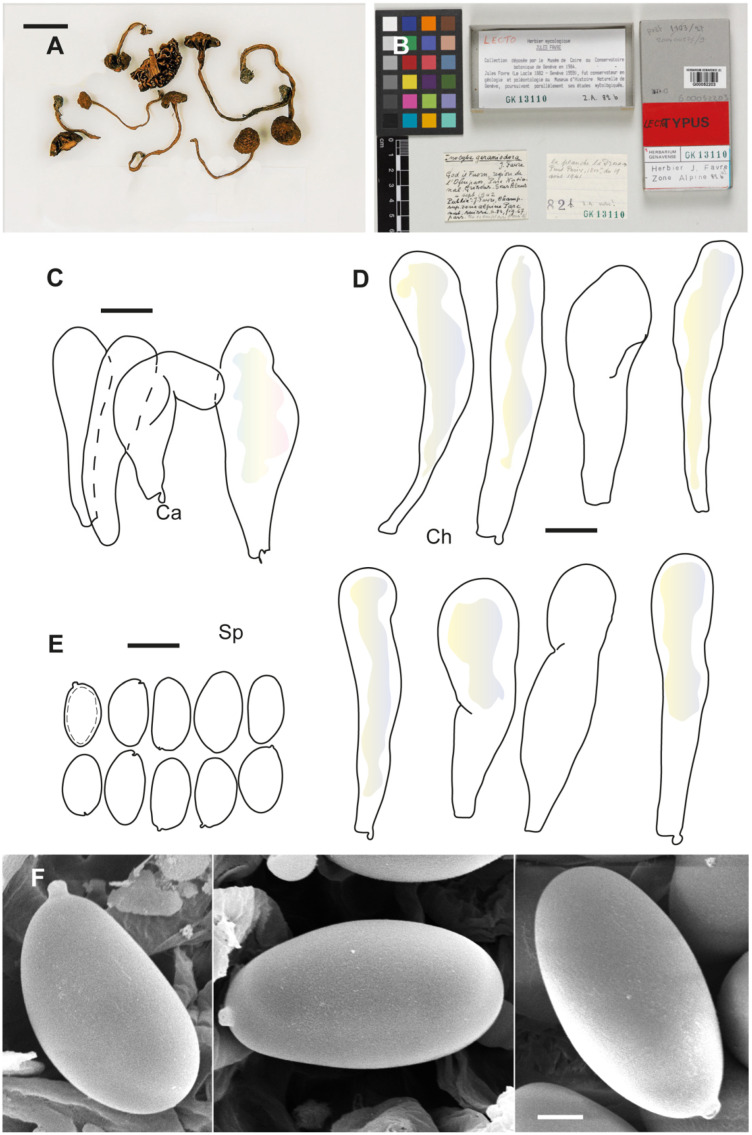
*Inosperma geraniodorum* Lectotype Herb. J. Favre Z.A.82b. (**A**,**B**) Voucher material and label. (**C**) Caulocystidia. (**D**) Cheilocystidia. (**E**) Basidiospores. (**F**) Spore SEMs. Scale bars: 10 mm (**A**); 10 µm (**C**–**E**); 2 µm (**F**).

Figure 11 and Figure 15C,D

≡ *Inocybe geraniodora* J. Favre, Ergebnisse der Wissenschaftlichen Untersuchungen des Schweiszerischen Nationalparks 5: 200, 1955. MycoBank No. 298912.

**Lectotype.** Switzerland, Graübunden, Parc National Grisons, Ofen Pass, God dal Fuorn: 46°39′42.78″ N, 10°12′42.66″ E, under *Alnus alnobetula*, 1850 m alt., 4 September 1942, Herb. J. Favre Z.A.82b (G 00052203), GenBank accession: ITS1 (PP431545), ITS2 (PP431549). Lectotype designated by Monthoux and Kuyper in Kuyper ([4]: 37). MBT 10013961.

**Description.** Favre [69], Kuyper [4], Senn-Irlet [80], Bizio [81], Bon [65], Breitenbach and Kränzlin [57].

**Additional microscopic examination of the lectotype of *I. geraniodora*.** *Basidiospores* (11.0–)11.7–15.5(–16.0) × 7.1–8.8(–9.2) µm, Sp_avg_ = 13.6 × 7.9 µm, Q = (1.4–)1.5–1.8(–1.9), Q_avg_ = 1.7 (n = 30), smooth, thick-walled (≈ 1 µm), mostly ellipsoid to broadly ellipsoid, hardly or not phaseoliform in profile, ochraceous brown. *Basidia* clavate, mostly four-spored, hyaline, often with intracellular red-brownish pigment. *Pleurocystidia* absent. *Cheilocystidia* 45.0–55.1(–60.0) × (9.5–)10.1–17.6(–18.1) µm, Ch_avg_ = 51.3 × 13.8 µm (n = 8), subcylindrical to subclavate or clavate, with rounded apex, hyaline, thin-walled, often with reddish-brown content. *Caulocystidia* present near apex, grouped in clusters, similar to cheilocystidia, 40.7–48.8 × 11.4–17.7 µm, Ca_avg_ = 44.4 × 13.8 µm. *Clamp connections* present.

**Distribution**. Its distribution is confirmed in the boreal and alpine regions of Europe and North America. In Europe, it has been found in the Alps (France, Italy and Switzerland), the Pyrenees (Spain), Finland, Iceland, Norway and Sweden. Three references deposited in GenBank match the lectotype of *I. geraniodorum*: KC965816 confirms its presence in Arctic Canada on Banks Island [82]; the other two are from Svalbard (Norway), JF304334 [83] and from Sweden, MH310767 [84]. There are no sequences in the UNITE database that match *I. geraniodorum*.

**Ecology.** *Inosperma geraniodorum* inhabits alpine and northern boreal ecosystems on calcareous soils, associated with various dwarf willows (*Salix reticulata*, *S. retusa*, etc.) and *Dryas octopetala*, and most probably with *Bistorta vivipara*. It can also develop in subalpine ecosystems at the upper limit of coniferous forests, between 1750 and 2000 m alt. [85]. The lectotype was collected in the subalpine level close to *Alnus alnobetula*, with which it can probably establish ectomycorrhizae.

**Etymology.** From the Latin *Geranium*, the plant which is commonly called pelargonium or geranium, and *odor* = smell, because its smell is reminiscent of this plant.

**Additional specimens examined.** Italy, Veneto, Belluno, Canale d’Agordo, Pian delle Comelle: 46°17′38″ N, 11°51′43″ E, under *Dryas octopetala* in sandy calcareous soil, 1827 m alt., 10 August 2008, leg. E. Bizio, EB 2008081002 (dupl. AH 56199), GenBank accession: ITS (PP431523). Veneto, Belluno, Cortina d’Ampezzo, Passo Falzarego: 46°30′29″ N, 12°01′50″ E, alpine scrubland in calcareous soil with *Salix retusa* and *Dryas octopetala*, 2238 m alt., 3 August 2019, leg. E. Bizio, EB 2019080304 (dupl. AH 46961), GenBank accession: ITS (PP431524). Norway, Oppland, Dovre, Grimsdalen: alpine heath with *Salix reticulata* on calcareous soil, 21 August 2012, leg. E. Larsson, EL 105-12 (GB 0243141), GenBank accession: ITS-LSU (OR823941). Spain, Cataluña (Catalonia), Lleida, Espot, Muntanya dels Estanyets: 42°32′37.92″ N, 1°4′21.45″ E, in a community of dwarf willows (*Salix reticulata*) and *Dryas octopetala*, in calcareous soil, 2240 m alt., 22 August 1999, leg. J. Llistosella, J. Vila, J. Girbal & F. Esteve Raventós, AH 25490 (dupl. JVG 990822-5). Sweden, Torne lappmark, Jukkasjärvi, Kopparåsen: alpine heath with *Dryas octopetala* and *Salix reticulata* on calcareous soil, 635 m alt., 17 August 2017, leg. E. Larsson, EL 156-17 (GB 0243140), GenBank accession: ITS-LSU (OR823942), RPB2 (PP092176). Lule lappmark, Jokkmokk, Padjelanta NP: alpine heath with *Dryas octopetala* and *Salix reticulata* on calcareous soil, 860 m alt., 14 August 2016, leg. H. Croneborg, EL 126-16 (GB 0243139), GenBank accession: ITS-LSU (OR823943), RPB2 (PP092175).

**Notes**. *Inosperma geraniodorum* can be recognised by the absence of a blue-greenish colour of the stipe, the slender habit (“mycenoid” type, according to Vellinga [25]), usually with a paraboloid to campanulate, often subumbonate pileus, the dark chocolate-brown colour of the basidiomata, the fibrillose to squamulose surface of the pileus (Figure 15C,D), and the large ellipsoid spores (Figure 11E,F). In young specimens, the typical smell of pelargonium is perceptible, although in some cases a fishy or cucumber component is present, especially in mature specimens. Similar in odour to *I. geraniodorum* is *I. veliferum*, which is slightly smaller and has a convex, not or hardly umbonate pileus, which is finely felted–fibrillose and initially covered with a persistent whitish veil. *Inosperma geminum*, with an odour and habitat similar to those of pelargonium, is another small and reddish-brown species, but it does not have a persistent veil and differs from *I. geraniodorum* and *I. veliferum* in its slightly smaller and narrower spores, with marked phaseoliform to naviculiform tendency in profile, and shorter cheilocystidia on average.

According to the data obtained from our phylogenetic analysis, the species most closely related to *I. geraniodorum* is *I. turietoense*. Both have a similar habit, with a slender stipe much longer than the diameter of the pileus, a brown to brownish-red colour, a poorly developed or absent veil, a pileus with a fibrillose scaly surface, and large spores. However, they differ in their ecological preferences, as *I. turietoense* is a nemoral species in mountainous areas, not reaching subalpine or alpine altitudes, it is larger in size, and the pileus is decorated with a characteristic appressed scaly, tabby ornamentation, which contrasts strongly with the background due to its darker colour. *Inosperma turietoense* does not have the characteristic pelargonium odour of *I. geraniodorum*.

Favre [69] first described *I. geraniodorum* based on several collections without designating a holotype (citing several syntypes). He also mentioned its occurrence in the upper subalpine zones of the areas visited [85]. The collection chosen by Kuyper [4] as the lectotype has been successfully sequenced and allows us to clarify its taxonomic position. In the herbarium of J. Favre, deposited in G, there are about twenty collections identified by the Swiss mycologist as *Inocybe geraniodora*. Apart from the lectotype, molecular data have not yet been obtained for any of them, so it is quite likely that some may correspond to other close or similar species (such as *I. veliferum*).

Favre’s original description of *I. geraniodorum* was based on the syntypes, and this fact can be confirmed by the various collections mentioned and drawn by him in the protologue. The study of the lectotype has shown spores slightly shorter than those given by Favre [14–18(–19.5) µm], but it cannot be ruled out that some of these collections have bisporic basidia (a common occurrence in high mountain collections), and consequently the basidiospores show some variability in length. However, we did not observe bisporic basidia in the lectotype, as the hymenial elements were collapsed. This is quite common in *Inosperma* specimens. In Favre’s iconography ([69]: 83, Figure 67) the macromorphological variability between different collections and their microscopic characteristics are clearly shown.

*Inocybe geraniodora* var. *gracilenta* was also introduced by Favre [69] but invalidly published. It was considered a variant of *I. geraniodora* in the alpine zone, characterised by its very small size, smaller spores and shorter cheilocystidia, 32–50 × 9–13 µm. The only existing collection in G was successfully sequenced, and the ITS was obtained. It corresponds to a species belonging to the Calamistratum group and is presented in this paper as a new species (see *I. gracilentum*). Favre [69] does not mention the odour of this variant, but, presumably because of its name, it should be like that of *I. geraniodorum* and therefore have a pelargonium component. Also, in Favre’s iconography ([69], pl VI, Figure 5), the specimens do not show a blue-greenish tinge at the base of the stipe, which could lead to misinterpretation (Favre’s iconography could bring to mind specimens of *I. geraniodorum*).

Favre [85] also described *Inocybe geraniodora* var. *depauperata*, collected from high mountain forests in Switzerland, under conifers (*Pinus* and *Larix*). It is a small species with a hirsute scaly pileus in the central zone, without significant odour, with large amygdaliform spores (15–17 × 7.5–9 µm) and long cheilocystidia (50–70 µm), generally with a capitate apex (12–19 µm diam.). Two collections exist in the Herbarium G. One of them (God Cumün) was successfully sequenced, and its phylogenetic placement demonstrates that it should be included in the Cervicolor group and not in the Calamistratum and Geraniodorum groups dealt with in this paper.

***Inosperma geminum* E. Larss. & Vauras, sp. nov.**

MycoBank No. 850670

**Figure 12:**
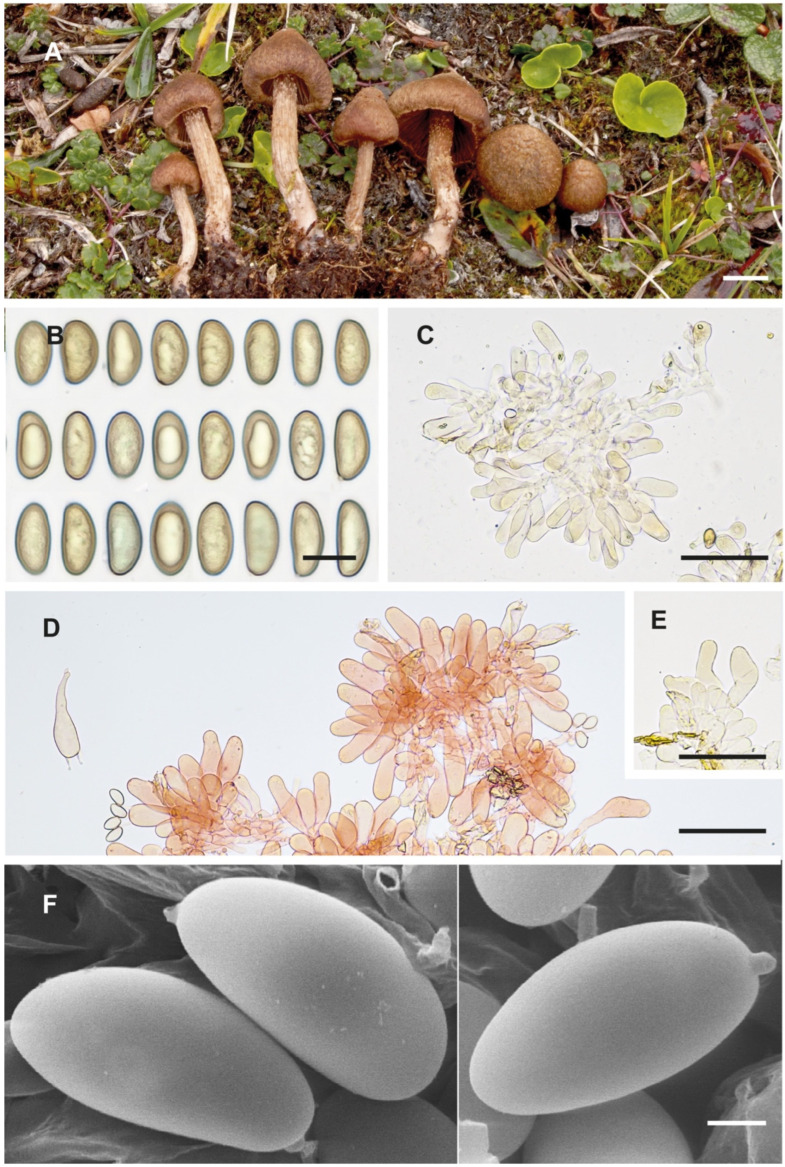
*Inosperma geminum* Holotype JV 31497 (TUR). (**A**) Basidiomata. (**B**) Basidiospores. (**C**) Cheilocystidia. (**D**) Cheilocystidia and basidia. (**E**) Caulocistydia. (**F**) Spore SEMs. Scale bars: 10 mm (**A**); 10 µm (**B**); 50 µm (**C**–**E**); 2 µm (**F**). Photograph (**A**) by J. Vauras.

Figure 12 and Figure 15A,B

**Diagnosis.** *Inosperma geminum* differs from other morphologically similar species, such as *I. geraniodorum* and *I. veliferum*, by having smaller basidiomata and narrower spores that are adaxially plane to slightly concave or subphaseoliform, often with a navicular appearance in lateral view. They differ in ITS sequence data and are phylogenetically distinct.

**Holotype.** Sweden, Lule lappmark, Jokkmokk, Padjelanta NP, Tuottar: alpine site, SW slope with *Dryas octopetala*, *Salix reticulata* and *S. herbacea*, on calcareous ground, 980 m alt., 13 August 2016, leg. J. Vauras, JV 31497 (TUR, isotypes in GB 0207615 and AH 56239), GenBank accession: ITS-LSU (OR823936), RPB2 (PP092174).

**Description.** *Pileus* 8–25 mm; when young, hemispherical, conical to obtusely conical with incurved margin; later, conico-convex to plano-convex, sometimes with a broad umbo. Surface dry, fibrillose to finely scaly; with age, squamulose at centre of the disc, ochraceous brown to reddish brown, velipellis ephemeral, pale. *Lamellae* rather sparse, broadly adnate to emarginate (L = 26–36), interspaced with lamellulae; at first, pale beige; with age, ochraceous brown; edge pale, fimbriate. *Stipe* 15–30 × 1.5–3 mm, dry, equal to slightly bulbous, pale ochraceous brown; later, concolourous with pileus, fibrillose to coarsely fibrillose; at apex, white, flocculose. *Context* ochraceous brown. *Odour* distinct of pelargonium but often also of fish. *Basidiospores* 11.8–12.5(–15.3) × 5.6–6.7(–7.4) Sp_awg_ = 12.5 × 6.7 µm, Q = 1.7–2.0, Q_awg_ = 1.9 (n = 150/5), smooth, ochraceous brown, variable in shape, ellipsoid to narrowly ellipsoid in face view, some adaxially plane to slightly concave, quite often subphaseoliform with a navicular appearance, apiculus small, ochraceous brown. *Basidia* 42–48 × 10–13 µm (n = 45/5), narrowly clavate, mainly four-spored, hyaline. *Pleurocystidia* absent. *Cheilocystidia* 20–48(–53) × 11–17(–20) µm (n = 45/3), pyriform, clavate or subcylindrical, hyaline, thin-walled, some with brownish content. *Caulocystidia* present near the apex, similar to cheilocystidia but generally shorter, abundant, 20–38 × 9–16 µm (n = 20/2). *Clamp connections* present.

**Distribution.** So far known from the alpine zone in Sweden and Norway. No additional sequence matching data were available in GenBank, but there is one specimen in the UNITE database that originates from Norway (UDB07673341|NOBAS 1542-15).

**Ecology.** It seems to be restricted to herb-rich alpine ecosystems on calcareous ground, associated with *Dryas octopetala* and *Salix reticulata*.

**Etymology.** Refers to the Latin word *geminum*, meaning twin, double, pair, resembling or similar to, because of its similarity to *I. veliferum* and *I. geraniodorum*.

**Additional specimens examined.** Norway, Oppland, Dovre, Kongsvoll: in subalpine *Betula* forest on calcareous soil, with *Salix reticulata*, 20 August 1986, leg. L. & A. Stridvall 86/107, GB 0064330, GenBank accession: ITS (OR823940). Sweden, Torne lappmark, Jukkasjärvi, Latnja: alpine cliff ecosystem on calcareous ground, with *Dryas octopetala*, 925 m alt., 6 August 2006, leg. E. Larsson, EL 63-06 (GB 0207619, dupl. AH 56241), GenBank accession: ITS-LSU (OR823936), RPB2 (PP092172). Torne lappmark, Jukkasjärvi, Orddajohka towards Vilgesgierdu: alpine cliff ecosystem on calcareous ground, with *Dryas octopetala*, 12 August 2017, leg. E. Larsson, EL 50-17 (GB 0207618), GenBank accession: ITS-LSU (OR823938), RPB2 (PP092173). Härjedalen, Storsjö, Svansjökläppen: alpine area with *Dryas otopetala* on calcareous ground, 17 August 2006, leg. E. Larsson, EL 106-06 (GB 0207617, dupl. AH 56240), GenBank accession: ITS-LSU (FN550945)—as *Inocybe geraniodora*. Jämtland, Frostviken, Raavre: alpine heath on calcareous soil with *Dryas octopetala* and *Salix reticulata* on calcareous ground, 820 m alt., 23 August 2019, leg. H. Croneborg 96-19, GB 0207616, GenBank accession: ITS-LSU (OR823939).

**Notes**. *Inosperma geminum* is very similar to both *I. geraniodorum* and *I. veliferum*. It is a small species characterised by an obtusely conical to plano-convex ochraceous-brown to reddish-brown pileus, fibrillose at the margin and distinctly squamulose at the centre of the pileus, with an ochraceous-brown stipe (Figure 12A and Figure 15A,B). It has a distinct odour of pelargonium and fish. It is phylogenetically most closely related to *I. veliferum*, but the two species form separate, distinctly supported clades (Figure 1). The two can also be separated based on differing ecologies and geographic distributions, and they differ in ITS sequence data by eight substitutions, five single, one of 2 bp, one of 4 bp and one of 7 bp insertion/deletion events. *Inosperma geminum* can be confused with *I. geraniodorum*, as they have similar habitat and their geographic distributions and ecologies overlap. *Inosperma geminum* seems to be rare and less common than *I. geraniodorum* and has on average smaller basidiomata and a smaller pileus diameter than the latter. The two can also be separated in terms of micromorphology, as *I. geminum* has, on average, slightly shorter spores (Figure 12B,F) and a larger Q_avg_ = 1.9 vs. 1.6.

***Inosperma turietoense* Pancorbo & Esteve-Rav., sp. nov.**

MycoBank No. 850671

**Figure 13:**
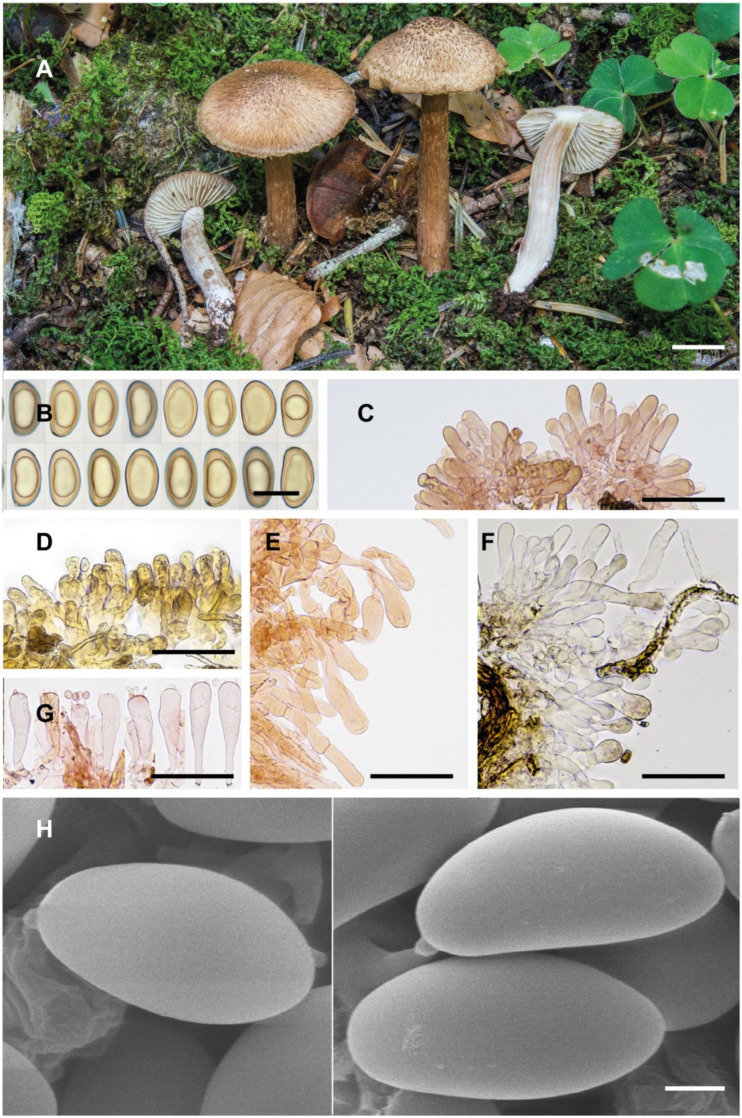
*Inosperma turietoense* Holotype AH 47710. (**A**) Basidiomata. (**B**) Basidiospores. (**C**) Cheilocystidia. (**D**) Laminar edge. (**E**,**F**) Caulocystidia. (**G**) Basidia. (**H**) Spore SEMs. Scale bars: 10 mm (**A**); 10 µm (**B**); 50 µm (**C**–**G**); 2 µm (**H**).

Figure 13 and Figure 15G,H

**Diagnosis.** *Inosperma turietoense* is similar to *I. geraniodorum*, but differs in its larger size, narrower spores and montane rather than alpine habitat. It also has a different odour, with no pelargonium component. *Inosperma veliferum* and *I. geminum* are smaller, grow in alpine ecosystems and have a typical pelargonium smell. The four species can also be distinguished by ITS sequence data.

**Holotype.** Spain, Aragón, Huesca, Torla, Ordesa National Park, Turieto Alto: 42°39′00″ N, 0°4′43″ W, in continental montane mixed forest of *Abies alba* and *Fagus sylvatica*, on calcareous soil, 1350 m alt., 26 August 2016, leg. F. Cervera, F. Serrano, F. Mateo, G. Sánchez, F. Tello, F. Pancorbo & F. Esteve-Raventós, AH 47710 (isotypes in FP 16082601 and GB 0266842), GenBank accession: ITS (PP431526), LSU (PP431541), RPB2 (PP478208).

**Description.** *Pileus* 20–30 mm, broadly hemispherical to convex when young, later conico-convex to plano-convex, centre subumbonate. Surface dry, fibrillose, finely scaly–fibrillose radially, appressed squamulose especially at centre, scales darker and contrasting with the background, appressed, not recurved, forming a delicate net and forming a virgate to tabby appearance, colour brown to reddish brown, velipellis not observed. *Lamellae* rather sparse, ventricose, narrowly adnate to annexed (L = 36–48), interspaced with lamellulae (l = 1–2); at first, whitish to pale beige; with age, ochraceous brown; edge pale, fimbriate. *Stipe* 50–70 × 5–8 mm, dry, equal to tapering downwards, straight to sinuose; at first, dirty white, then pale ochraceous brown; later, concolourous with pileus; fibrillose to coarsely fibrillose; at apex, white, flocculose. *Context* ochraceous brown, reddening along the stipe and pileus. *Odour* distinct and complex when cut, earthy to mouldy, sometimes mixed with some aromatic component, reminiscent of a mixture of *Inosperma cervicolor*/*bongardii* smells. *Basidiospores* smooth, ochraceous brown, (9.8–)10.4–13.4(–14.7) × (6.2–)6.4–7.3(–7.6) µm, Sp_avg_ = 12.0 × 6.8 µm, Q = (1.45–)1.50–1.94(–2.02), Q_avg_ = 1.7 (n = 209/2), smooth, ellipsoid to subamygdaliform, sometimes subphaseoliform, in lateral view. *Basidia* long, (46.1–)51.5–61.6(–64.2) × (11.5–)11.8–14.0(–16.3) µm, B_avg_ = 53.4 × 13.0 µm, narrowly clavate, mainly four-spored, hyaline, some with intracellular brown pigment. *Pleurocystidia* absent. *Cheilocystidia* (31.2–)33.7–56.2(–64.5) × (7.5–)8.4–12.8(–15.0) µm, Ch_avg_ = 43.5 × 10.7 µm (n = 82/2), subcylindrical with rounded apex, subclavate, sometimes subcapitate, base (multi)septate, thin-walled, some with brownish content. *Caulocystidia* present near apex, similar to cheilocystidia, multiseptate, but generally larger than hymenia cystidia, abundant, (26.1–)27.9–78.7(–133.3) × (6.8–)8.0–16.8(–17.1) µm, Ca_avg_ = 53.3 × 12.4 µm, (n = 42/2). *Clamp connections* present.

**Distribution.** So far, known from the type locality in the Central Pyrenees of Spain.

**Ecology.** On calcareous soils in montane continental areas, in mixed beech (*Fagus sylvatica*) and fir (*Abies alba*) forests, in the *Buxo*–*Fagetum* community [86].

**Etymology.** Refers to the locality where it was found, called Turieto Alto.

**Additional specimens examined.** Spain, Aragón, Huesca, Torla, Turieto Alto: 42°38′56″ N, 0°4′12″ W, 1352 m alt., mixed forest of *Fagus sylvatica* and *Abies alba* in calcareous soil, 30 August 2015, leg. G. Sánchez, M.A. Ribes, F. Pancorbo & F. Esteve-Raventós, AH 47669 (dupl. FP 15083005), GenBank accession: ITS (PP431525), LSU (PP431540).

**Notes**. *Inosperma turietoense* has certain morphological similarities and is phylogenetically closely related to *I. geraniodorum*. The basidiomes are of medium size and slender appearance, but larger than those of *I. geraniodorum* (Figure 13A and Figure 15G,H). The ellipsoidal spores (Figure 13B,H) are similar in both, although on average slightly narrower in *I. turietoense* (13.7 × 7.9 µm vs. 12.0 × 6.8 µm). It also differs in several notable diagnostic characters: the tabby scaly appearance of the pileus centre, with darker adpressed and delicate scales, the different odour without traces of pelargonium, and a more continental, temperate, montane habitat. In the collections studied, *I. turietoense* has a typically attenuated stipe towards the base, and the reddening of the flesh and surface of the stipe is noticeable. Collections made in situ have led us to make an approximate identification with a member of the Cervicolor group, whose species are phylogenetically distant and show some differences in pileus appearance, but it gives an idea of the first impression of the new species. *Inosperma veliferum* and *I. geminum* have smaller basidiomata with a distinct whitish velipellis, at least when young, a pure pelargonium smell in young basidiomes, and boreal–alpine distribution.

***Inosperma veliferum* (Kühner) Matheny & Esteve-Rav., Mycologia 112(1): 105, 2019.**

MycoBank No. 830407

**Figure 14:**
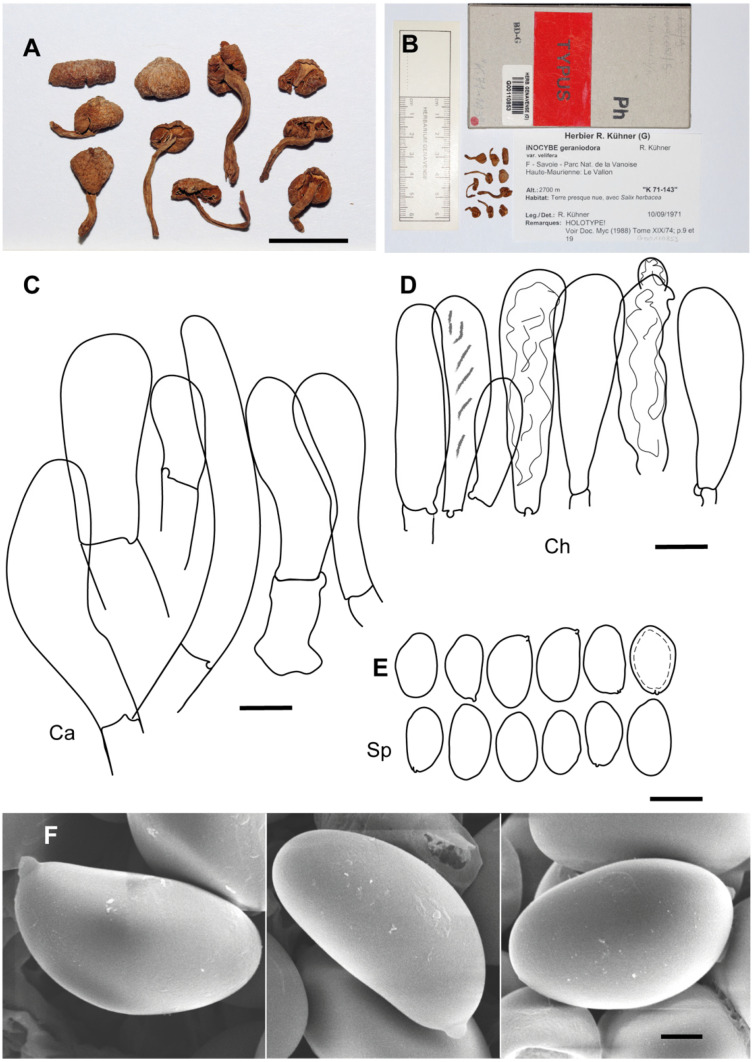
*Inosperma veliferum* Holotype Herb. R. Kühner 71-143 (G 00110853). (**A**,**B**) Voucher material and label material. (**C**) Caulocystidia. (**D**) Cheilocystidia. (**E**) Spores. (**F**) Spore SEMs. Scale bars: 10 mm (**A**); 10 µm (**C**–**E**); 2 µm (**F**). Photographs (**A**,**B**) by J.C. Zamora.

**Figure 15:**
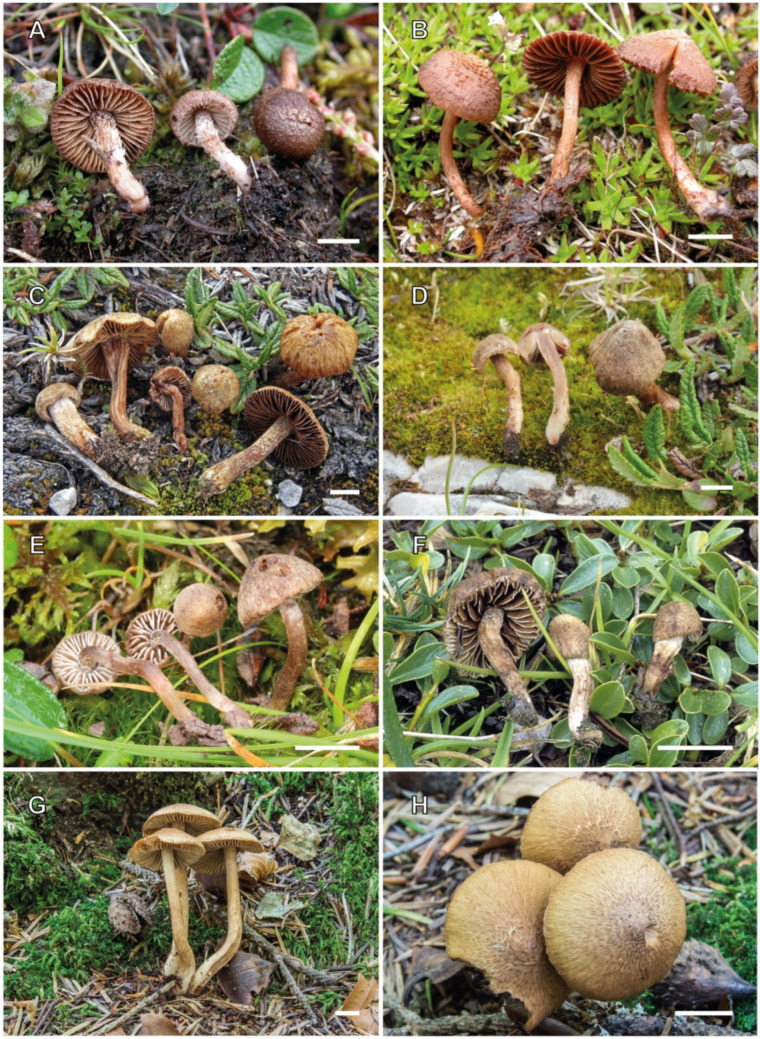
Basidiomes of the species of the Geraniodorum group. (**A**) *Inosperma geminum* EL 63-06 (SWE). (**B**) *I. geminum* EL 106-06 (SWE). (**C**) *I. geraniodorum* EB 20100823 (ITA). (**D**) *I. geraniodorum* AH 46961 (ITA). (**E**) *I. veliferum* AH 46962 (ITA). (**F**) *I. veliferum* AH 56198 (ITA). (**G**,**H**) *I. turietoense* AH 47669 (ESP). Scale bars: 10 mm (**A**–**H**). Photograph (**C**) by C. Zoldan.

Figure 14 and Figure 15E,F

≡ *Inocybe geraniodora* var. *velifera* Kühner, Documents Mycologiques 19(74): 19, 1988. MycoBank No. 135089.

≡ *Inocybe velifera* (Kühner) Bon, Bulletin Trimestriel de la Fédération Mycologique Dauphiné-Savoie 37(144): 78, 1997. MycoBank No. 436881.

**Holotype.** France, Savoie, Parc National de la Vanoise, Haute-Maurienne, Le Vallon: 45°25′42″ N, 6°24′17″ E, on almost bare ground with *Salix herbacea*, 2700 m alt., 10 September 1971, Herb. R. Kühner 71-143 (G 00110853), GenBank accession: ITS (PP431520).

**Description.** Kühner [77], Bon [65].

**Additional microscopic examination of the holotype of *I. geraniodora* var. *velifera.*** *Basidiospores* (10.8–)11.5–14.5(–15.5) × 7.1–9.0(–9.5) µm, Sp_avg_ = 13.2 × 8.0 µm, Q = (1.38–)1.44–1.74(–1.80), Q_avg_ = 1.6 (n = 21), smooth, thick-walled (≈ 0.7 µm), ellipsoid to broadly ellipsoid, ochraceous brown. *Basidia* mostly collapsed, clavate, four-spored, hyaline, often with intracellular brown pigment. *Pleurocystidia* absent. *Cheilocystidia* (26.0–)30.5–50.9(–51.4) × (9.6–)10.0–14.2(–14.5) µm, Ch_avg_ = 42.7 × 12.0 µm (n = 7), variable in shape, subcylindrical, clavate, sublageniform, sometimes subcapitate, thin-walled, some with brownish content. *Caulocystidia* present near apex, in clusters, similar to cheilocystidia, numerous, (22.9–)25.6–67.5(–72.1) × (9.8–)10.3–18.1(–18.8) µm, Ca_avg_ = 44.7 × 13.6 µm, (n = 7). *Clamp connections* present.

**Distribution**. *Inosperma veliferum* is distributed in central and southern alpine areas of the European continent. Except for one matching sequence, we are not aware of any more collections or deposited sequences from boreal areas in either GenBank or UNITE databases. Its current distribution includes France [65,77], Italy (GenBank JF908117—as *I. geraniodora*) and Spain ([87]—as *I. geraniodora* var. *geraniodora*).

**Ecology.** It seems to be a strictly alpine species that thrives at high altitudes. It was originally described in association with *Salix herbacea*, a plant that prefers acidic soils, although it can invade snow patches on calcareous substrates [88]; the collections studied in Italy and Spain were found in mats of *Salix retusa*, *S. reticulata* and *Dryas octopetala* on calcareous soils. All collections were found above 2100 m alt.

**Etymology.** Derived from the Latin *velifer*, from *velum* = veil and *-fer* = to carry, referring to the presence of a veil.

**Additional specimens examined.** Italy, Trentino-Alto Adige, Sen Jan di Fassa (TN), Rifugio Vallaccia: in calcareous soil with *Salix retusa*, *S. reticulata* and *Dryas octopetala*, 2355 m alt., 14 August 1994, leg. E. Bizio, MCVE 4485, GenBank accession: ITS (JF908117)—as *I. geraniodora*. Veneto, Falcade, Gruppo Marmolada, Passo col Becher: 46°23′54″ N, 11°52′04″ E, in calcareous soil in *Salix retusa* and *S. reticulata* scrub, 2292 m alt., 9 August 1993, leg. E. Bizio, EB 1993080906 (dupl. AH 56198 and MCVE 20884), GenBank accession: ITS (PP431519), LSU (PP431539). Veneto, Belluno, Falcade, Forcella Venegia: 46°19′55″ N, 11°46′26″ E, alpine scrub with *Salix retusa*, *S. reticulata*, *Dryas octopetala*, *Bistorta vivipara* and *Kalmia procumbens*, in calcareous soil, 2250 m alt., 10 August 2019, leg. E. Bizio, AH 46962, GenBank accession: ITS (PP431521). Spain, Aragón, Huesca, Hoz de Jaca, Sierra de Tendenera, Peña Sabocos: 42°41′14.32″ N, 0°15′40.77″ W, in calcareous bare soil with scattered scrubs of *Salix reticulata* and *Dryas octopetala*, 2180 m alt., 27 August 1996, leg. F. Arenal, E. Horak & F. Esteve Raventós, AH 21346, GenBank accession: ITS (PP431522).

**Notes**. *Inosperma veliferum* is morphologically very close to *I. geminum* and *I. geraniodorum*. These species have initially a pure pelargonium smell and lack blue-greenish pigments in the basidiomata. In addition, the appearance of the surfaces of the pileus and stipe differs markedly between the members of the Geraniodorum group and those of the Calamistratum group, the latter being characterised by a distinct scaly or even coarsely scaly to squarrose appearance of their surfaces. The species of the Calamistratum group show spores that are more elongated and narrower, ellipsoid to subcylindrical or subphaseoliform. Only *I. subhirsutum* seems to be an exception, as some collections (including the holotype) show broadly ellipsoidal spores and more fibrillose–woolly to squamulose surfaces.

*Inosperma geraniodora* var. *velifera* was described by Kühner [77] from the French Alps. Bon [65] raised it to the rank of a separate species because of the presence of the characteristic veil and the lower Q of the spores. It is recognisable by its small size and the more obtuse and convex appearance of the pileus, which is not or hardly umbonate and never has a squamulose surface, but is smooth to fibrillose (Figure 14A and Figure 15E,F). A good diagnostic feature is the presence of an abundant whitish veil, especially in young or unwashed specimens, which totally or partially camouflages the reddish-brown colour of the pileus. In *I. geraniodorum* and *I. geminum*, the veil may be present on the pileus disc in young stages, but in no case is it so abundant or developed, and both have a subsquamose pileus centre. Because of its small size, *I. veliferum* can be confused with *I. geminum*, the species to which it appears to be the most closely related phylogenetically. The pileus of the latter is sometimes more campanulate and subumbonate and is distinctly squamulose at the centre, and its spores are narrower (13.2 × 8.0 µm vs. 12.5 × 6.7 µm), with a marked subphaseoliform to naviculiform tendency in profile. So far, *Inosperma geminum* has been found exclusively in the alpine zone of the Scandinavian mountains, whereas *I. veliferum* seems to be restricted to the alpine areas of central Europe and the Pyrenees. However, *I. geraniodorum* is widespread in both alpine and boreal regions and can also be found at subalpine altitudes between 1800 and 2000 m [85]. Comparative studies of the size and shape of spores and cystidia of the samples of *I. geraniodorum* and *I. veliferum* studied have not shown any remarkable or distinct microscopic differences between the two for diagnostic use. Both species have similar average dimensions in spore size, as well as size and shape of the cystidia. The macroscopic appearance of *I. geraniodorum* is slightly different, producing darker and generally larger basidiomes, often with a squamulose conical to subumbonate pileus, and the veil, if present, is more diffuse and transient.

**Provisional key for the recognition of the European species of the genus *Inosperma* (Calamistratum/Geraniodorum Groups)**

1- A characteristic blue-green or grey-green colour at the base of the stipe (young, not water-soaked specimens should be observed; in some collections of *I. subhirsutum*, these colours may sometimes be absent). Spores with W_avg_ = 5–7 µm—Calamistratum Group............................................................................................................................................... **2**
1′- Blue-green colours absent. Spores with W_avg_ = 6.7–8.0 µm—Geraniodorum Group.............................................................................................................................................. **6**
2- Pileus and stipe surfaces strongly hirsute–squamose to squarrose. Habitat Atlantic/continental, nemoral to boreo-nemoral, under conifers, *Betulaceae*, *Fagaceae* or mixed coniferous/broadleaved forests..................................................................................................... **3**
2′- Pileus and stipe surfaces hirsute–fibrillose, scales not so bristly, more flattened, often tessellated and woolly in appearance, initially covered by an ochraceous veil (observe young specimens). Habitat boreal, alpine or Arctic, sometimes at subalpine levels............................................................................................................................................... **4**
3- Pileus and stipe surfaces (observe young specimens) strongly bristly, squarrose. Scales distributed over most of the surface of the pileus at maturity, individualised, rarely welded together. Spores long, ellipsoidal in frontal view, phaseoliform in profile (W = 55.5 µm, Q_avg_ = 2), cheilocystidia mostly subcylindrical to claviform, often two- to three-septate at base, <40 µm in length.................................................................................... ***I. calamistratum***
3′- Pileus surface typically decorated in the centre with broad, often welded scales (tessellated–pyramidal appearance) at maturity, contrasting with the squamulose–fibrillose to smooth margin. Spores ellipsoidal in frontal view, phaseoliform in profile (W = 5.5–6 µm, Q_avg_ = 1.7), cheilocystidia broadly claviform to subspheropedunculate, more rarely subcylindrical, <45 µm in length............................................................... ***I. neohirsutum***
4- Spores very elongate, subcylindrical in frontal view, phaseoliform in lateral view, Q_avg_ = 2.2, cheilocystidia mostly cylindrical to broadly claviform, 40–60(–65) µm long. In boreal and alpine areas, on both acidic and calcareous soils, together with *Salix*, *Dryas*, *Betula nana* and *Bistorta*, sometimes also occurring in montane/subalpine and hemiboreal forests under conifers................................................................................................................. ***I. praetermissum***
4′- Spores different.............................................................................................................................. **5**
5- Spores long, ellipsoid in frontal view, ellipsoid–subphaseoliform in lateral view, Q_avg_ = 1.8, cheilocystidia typically short, mostly pyriform to broadly claviform, <40 µm long. In alpine environments and calcareous soils, under *Salix*, *Dryas* and *Bistorta*....................................................................................................................... ***I. gracilentum***
5′- Spores broadly ellipsoidal to subovoid in frontal view, ellipsoidal and hardly to slightly subreniform in lateral view, Q_avg_ = 1.6, cheilocystidia 35–50 µm, versiform, mostly claviform, broadly claviform to subcylindrical. Pileus and stipe scaly–fibrillose to woolly–fibrillose (“Mallocybe” appearance). Stipe with fibrillose to lanose surface. In alpine and boreal areas, in shrubland associated with *Salix*, sometimes with *Dryas*, *Betula nana* and *Bistorta*, less often associated with *Salix* trees in montane–subalpine coniferous forests (Alps)....................................................................................................... ***I. subhirsutum*** complex
6- Medium–small to small species, with reddish-brown pileus and stipe, with fibrillose scales concolourous to the pileus or pileus smooth–fibrillose, covered with a distinctive whitish veil when young. In alpine, subalpine and boreal areas................................................................................................................................................. **7**
6′- Medium-sized species, reminiscent of *I. cervicolor*/*subrubescens* in appearance, with brown to hazel pileus, decorated in the central area with delicate, darker and applied scales (brindled appearance). Cystidia subcylindrical, sometimes with subcapitate apex. Spores broadly ellipsoidal in frontal and lateral view, Q_avg_ = 1.7. In mixed montane *Abies*/*Fagus* forests on calcareous soils. Known to date from the Spanish Pyrenees..................................................................................................................... ***I. turietoense***
7- Species with broadly ellipsoid to ellipsoid spores in frontal and lateral view, Q_avg_ = 1.6–1.8..................................................................................................................................................... **8**
7′- Spores ellipsoid in frontal view, navicular–phaseoliform in lateral view, Q_avg_ = 1.9, cystidia versiform. Pileus typically squamose at the centre. In calcareous soils associated with *Salix* and *Dryas*. Species of exclusively boreal and alpine distribution, known so far from Scandinavia.......................................................................................................... ***I. geminum***
8- Pileus convex and obtuse at maturity; when young, covered by a manifest whitish veil on a smooth to finely fibrillose brownish ground (see unwashed specimens). Flesh reddish at the stipe cortex, not becoming very dark upon age. Spores broadly ellipsoidal to subovoid in both frontal and lateral view. In alpine areas, under *Salix* and *Dryas*, on both calcareous and acidic soils.............................................................................................................. ***I. veliferum***
8′- Pileus convex–campanulate at maturity, becoming scaly–fibrillose (sometimes bristly) in the centre, not covered by an apparent veil, or, if present, very ephemeral. Flesh dark reddish in mature basidiomata. Spores ellipsoid, broadly ellipsoid to subovoid in both frontal and lateral views, not or hardly subphaseoliform in profile. In alpine and boreal areas, associated with *Dryas*, *Salix* and *Bistorta*, but also in the upper subalpine level (Alps), probably associated with *Alnus alnobetula*........................................... ***I. geraniodorum***

## 4. Discussion

Organoleptic characters have historically had great importance in the recognition of *Inosperma* species. Contrary to what has been assumed in the past, members of *Inosperma* can be distinguished based on their morphological and ecological characteristics, and their identification is not so dependent on the interpretation of organoleptic characters. In the case of *Inosperma*, it is relatively common to collect specimens with certain morphological characteristics that are difficult to interpret, mostly due to the influence of environmental factors and the ageing of the basidiomes. These factors can modify the external appearance of certain characters (colour, size, appearance of the basidiomata surfaces, or redness), as well as the perceived odour. For *Inosperma*, it is particularly advisable to collect specimens in good and fresh condition and at different stages of development, which makes it easier to interpret these changes. In some collections, the bluish-green colour of the stipe base, so characteristic of the Calamistratum group, can be diminished or even disappear; in the same way, the reddening and odour of the specimens can undergo significant changes within a few hours. These changes greatly complicate the species determination if they are overestimated.

Our phylogenetic analyses inferred from the ITS-LSU-RPB1-RPB2 regions recovered nine clades containing samples obtained from European material and highly congruent with respect to morphological characters. All clades but one received phylogenetic support in at least one of the analyses. The *Inosperma subhirsutum* clade, however, did not receive support. The high sequence divergence within this clade and the high amount of missing data may have contributed to the lack of phylogenetic confidence. Our analyses support a high rate of continental endemicity within the *Calamistratum* group, as all the species seem to be restricted to a single continent, with the only—judging from the data available to date—exception of *I. praetermissum*, which appears to be present in North America (PBM 1105, WTU) as well. The fact that the backbone of the tree is not supported may be due to a high amount of missing data.

Our study demonstrates that the Calamistrata and Geraniodora groups are more diverse than previously considered in Europe and that several species have been subsumed under the names *I. calamistratum* and *I. geraniodorum*. The nine species present in Europe can be distinguished generally through morphological characters, at least when good, fresh, not weathered material is available. The main taxonomic informative characters that allow for species recognition are pileus and stipe scaliness, spore shape, and cystidial shape and size. Some species show a rather high sequence divergence in the ITS region (*I. subhirsutum* and *I. praetermissum*) and may comprise more than one evolutionary lineage. Nevertheless, obtaining a more complete dataset of protein-marker regions would be necessary to further test this possibility. We hope that this study contributes to an increased interest in and provides an updated identification guide for the Calamistratum and Geraniodorum groups.

## Data Availability

All of the data that support the findings of this study are available in the main text.
